# Co-expression of low-risk HPV E6/E7 and EBV LMP-1 leads to precancerous lesions by DNA damage

**DOI:** 10.1186/s12885-021-08397-0

**Published:** 2021-06-10

**Authors:** Karina Uehara, Yasuka Tanabe, Shintaro Hirota, Saki Higa, Zensei Toyoda, Kiyoto Kurima, Shinichiro Kina, Toshiyuki Nakasone, Akira Arasaki, Takao Kinjo

**Affiliations:** 1 Division of Morphological Pathology, Department of Basic Laboratory Sciences, School of Health Sciences, University of the Ryukyus, 207 Uehara, Nishihara, Okinawa, 903-0215 Japan; 2 Department of Oral and Maxillofacial Functional Rehabilitation, Graduate School of Medicine, University of the Ryukyus, 207 Uehara, Nishihara, Okinawa, 903-0215 Japan; 3 Neurobiology Research Unit, Okinawa Institute of Science and Technology Graduate University, 1919-1 Tancha, Onna, Okinawa, 904-0412 Japan; 4 Molecular Pharmacology and Oncology, Gunma University, Maebashi, Gunnma 371-8511 Japan

**Keywords:** Low-risk HPV, High-risk HPV, EBV LMP-1, Co-expression, Precancerous lesion

## Abstract

**Background:**

Low-risk human papillomavirus (HPV), such as types 6 and 11, is considered non-oncogenic, but these types have been detected in oral cancer tissue samples, suggesting their possible involvement in oral carcinogenesis. Because double infection of high-risk HPV and Epstein-Barr virus (EBV) is known to be involved in oral carcinogenesis, we hypothesized that low-risk HPV and EBV co-infection can transform the oral cells. To verify our hypothesis, we evaluated the transformation activity of cell lines expressing both low-risk HPV E6/E7 and EBV LMP-1.

**Methods:**

We transduced HPV6, 11 and 16 *E6/E7* genes and EBV *LMP-1* gene into primary mouse embryonic fibroblasts. The cell lines were examined for indices of transformation activity such as proliferation, induction of DNA damage, resistance to apoptosis, anchorage-independent growth, and tumor formation in nude mice. To evaluate the signaling pathways involved in transformation, NF-κB and p53 activities were analyzed. We also assessed adhesion signaling molecules associated with anchorage-independent growth such as MMP-2, paxillin and Cat-1.

**Results:**

Co-expression of low-risk HPV6 E6 and EBV LMP-1 showed increased cell proliferation, elevated NF-κB activity and reduced p53 induction. Moreover, co-expression of low-risk HPV6 E6 and EBV LMP-1 induced DNA damage, escaped from apoptosis under genotoxic condition and suppression of DNA damage response (DDR). Co-expression of low-risk HPV11 E6/E7 and EBV LMP-1 demonstrated similar results. However, it led to no malignant characteristics such as anchorage-independent growth, invasiveness and tumor formation in nude mice. Compared with the cells co-expressing high-risk HPV16 E6 and EBV LMP-1 that induce transformation, co-expression of low-risk HPV6 E6 and EBV LMP-1 was associated with low MMP-2, paxillin and Cat-1 expression.

**Conclusions:**

The co-expression of low-risk HPV E6/E7 and EBV LMP-1 does not induce malignant transformation, but it allows accumulation of somatic mutations secondary to increased DNA damage and suppression of DDR. Thus, double infection of low-risk HPV and EBV could lead to precancerous lesions.

**Supplementary Information:**

The online version contains supplementary material available at 10.1186/s12885-021-08397-0.

## Background

Human papillomavirus (HPV) is an oncogenic virus that causes uterine cervical, oral and anal cancers [1]. More than 200 HPV types with DNA sequence variations have been identified to date. In general, HPV has been roughly classified as high-risk or low-risk HPV, based on their association with malignant or benign tumors, respectively [2]. High-risk HPV such as HPV16 and HPV18 are the major oncogenic types detected in uterine cervical and oral cancers, whereas low-risk HPV such as HPV6 and HPV11 is related to benign lesions such as genital warts and papilloma [1].

HPV-encoded *E6* and *E7* genes are viral oncogenes that have a crucial role in cell transformation. In uterine cervical cancers, *E6* and *E7* from high-risk HPV are integrated into the genome, and their expression inactivates and degrades tumor suppressors p53 and pRb, respectively [1]. By contrast, low-risk HPV *E6* and *E7* gene expression only weakly inactivates p53 [3] and pRb [4]. Consequently, low-risk HPV is considered to have a lower transformation activity.

On the other hand, low-risk HPV such as HPV6 and HPV11 have been detected in oral cancer tissue samples, suggesting a possible involvement in oral carcinogenesis [5, 6]. However, it remains uncertain whether low-risk HPV actually causes oral cancer.

Double infection of HPV and Epstein-Barr virus (EBV) is known to be associated with oral cancer progression [7–9]. EBV is an oncogenic virus associated with nasopharyngeal, gastric cancers and Burkitt lymphoma [10, 11]. *LMP-1* is an EBV-encoded oncogene with the ability to transform EBV-infected B cells [12, 13] and human epithelial cells when co-expressed with Bcl-2 [14].

We previously reported that co-expression of high-risk HPV16 E6 and EBV LMP-1 transforms primary mouse embryonic fibroblasts (MEFs) [15]. Since double infection of high-risk HPV and EBV is involved in oral carcinogenesis, we hypothesized that low-risk HPV could lead to changes in oral cells if the cells are co-infected with EBV. However, whether such co-infection would actually lead to oncogenesis is unknown. In this study, we examined transformation activity of MEFs co-expressing low-risk HPV6/11 E6/E7 and EBV LMP-1, comparing with those co-expressing high-risk HPV16 E6 and EBV LMP-1. We also observed whether injecting these MEFs into nude mice developed tumors.

## Methods

### Cell lines and cell culture

Primary MEFs (CF-1) were purchased from American Type Culture Collection and cultured in Dulbecco’s Modified Eagle Medium (Nissui) with 10% fetal bovine serum (Wako). The cells were incubated at 37 °C in 5% CO_2_.

### Plasmids

The genes HPV6 *E6*, HPV6 *E7*, HPV11 *E6*, HPV11 *E7*, HPV16 *E6* and HPV16 *E7* were amplified by PCR containing restriction enzyme sites. These PCR products were digested with restriction enzymes, Bgl II and Xho I (New England Biolab), and ligated to pMSCV-*puro* vector (Clontech). Plasmids expressing EBV *LMP-1*, (i.e. pMSCV-*neo*-*LMP-1*) were prepared as previously described [15]. p*NF-κB*-*TA*-*luc*, p*p53*-*TA*-*luc*, p*β-gal*-basic (Clontech) and pcDNA3.1/*Zeo* (+) (Invitrogen) were also used for assays.

### Viral gene transduction via retrovirus

The procedure for retrovirus production and the construction of viral protein expressing MEFs have been described previously [16]. Briefly, the plasmids pMSCV-*puro*-*6E6*, pMSCV-*puro*-*11E6*, pMSCV-*puro*-*11E7* and pMSCV-*neo*-*LMP-1* were transfected into packaging cell lines PT-67 (Clontech) using Lipofectamine 2000 (Thermo Fisher Scientific) and incubated for 48 h. Retroviruses harboring the various HPV and/or EBV genes were generated, and CF-1 cells were then infected with the viruses. After retroviral infection, the cells were selected using 3 μg/ml puromycin or 150 μg/ml geneticin. Each viral gene expression was confirmed by RT-PCR (Supplemental Figure S1).

### RT-PCR

RNA was extracted from 1 × 10^6^ cells using total RNA isolation (Macherey-Nagel). One μg of total RNA was treated with DNase I Amplification Grade (Thermo Fisher Scientific), followed by reverse transcription using PrimeScript II 1st strand cDNA Synthesis Kit (Takara). Aliquots of the resulting cDNA were used as a template in PCR with primers shown in Supplemental Table S1.

### MTT (methyl thiazolyl tetrazolium) assays

The cells were seeded in 96-well plate at 1000 cells/well. The cell numbers were counted by adding cell count reagent SF (Nakalai Tesque) with the reaction performed at 37 °C for 90 min. Absorbance at 450 nm was measured by a micro plate reader (SH-1000, Corona).

### TdT-mediated dUTP-biotin nick end labeling (TUNEL) staining

Apoptotic cells were detected by TUNEL staining under genotoxic conditions. The cells were seeded on chamber slides (4 well SLIDE and CHANBER sterilized, Watson) at 5 × 10^4^ cells/well, treated with 125 μM H_2_O_2_ and then fixed in 4% paraformaldehyde for 30 min. After washing with PBS, TUNEL staining was performed using in situ Apoptosis Detection kit (Takara). The slides were treated with ProLong Gold Antifade Mountant with DAPI (Thermo Fisher Scientific).

### Immunocytochemistry

Similar as for TUNEL staining, the cells were seeded in chamber slides, and fixed in 4% paraformaldehyde. The fixed cells were treated with 0.5% TritonX-100 (Wako), and washed in PBS. After blocking with 1% BSA (Wako), the cells were incubated with primary antibodies (shown in Supplemental Table S2) for 60 min followed by Alexa Fluor 594 (Thermo Fisher Scientific) for 40 min. The slides were treated with ProLong Gold Antifade Mountant with DAPI (Thermo Fisher Scientific).

### Western blotting

Total protein was extracted from 1.0 × 10^6^ cells using SDS buffer containing protease inhibitor cocktail (Sigma-Aldrich). Ten μg of extracted protein was electrophoresed on 10% SDS-PAGE and transferred to polyvinylidene difluoride membranes (GE Health Care Life Sciences). The protein-bonded membranes were blocked with 5% non-fat milk or 5% BSA (Wako) for 60 min. After washing in TBS-T, the membranes were incubated with primary antibodies (shown in Supplemental Table S3). The signals were detected using Amersham ECL Prime (GE Health Care Life Sciences). Signal density was calculated by Image J software (National Institute of Health).

### Luciferase reporter assays

One day before analysis, the cells were seeded on a 96-well plate at 1.0 × 10^5^ cells/well. The cells were transfected with p*NF-κB*-*TA*-*luc* or p*p53*-*TA*-*luc* (100 ng), pcDNA3.1/ *Zeo* (+) (100 ng) and p*β-gal*-basic (50 ng) using Lipofectamine 2000 (Thermo Fisher Scientific) for 24 h. Cell lysates were assayed using a reporter assay system (Promega), and luciferase activity was measured by a Glomax 96 microplate luminometer (Promega). The lysates were incubated at 48 °C for 60 min, and assayed using the Galacto-Light Plus β-Galactosidase Reporter Gene Assay System (Applied Biosystem). Relative activities were calculated using β-galactosidase activity as an internal control.

### Soft agar colony formation assays

A 0.75% base agar containing DMEM, FBS, penicillin and streptomycin was added to 6-well plates. Subsequently, top agar (0.36% agar, DMEM, FBS, penicillin and streptomycin) containing 2.0 × 10^5^ cells was added onto the base agar. The cells were cultured for 4 weeks.

### Cell invasion assays

A total of 1.0 × 10^5^ cells were cultured with low serum medium (DMEM containing 0.5% BSA, 2 mM CaCl_2_ and 2 mM MgCl_2_) at 37 °C for 6 h and seeded onto a polycarbonate membrane. Invasive cells were detected by a CytoSelec 24-Well Cell Invasion Assay Basement Membrane, Colorimetric Format (Cell Biolabs).

### Comet assays

A total of 1.0 × 10^5^ cells were mixed with molten LMAgarose and 50 μl was placed on CometSlide (Trevigen). The slides were incubated with lysis solution (Trevigen) at 4 °C for overnight. Electrophoresis was performed at 100 V for 40 min. The electrophoresed slides were stained with SYBR® Green for 30 min in the dark. Comet length was measured using Image J (NIH).

### Animal experiments

Animal experiments were done in accordance with the guidelines for animal treatment, housing, and euthanasia of the Animal Experiment Committee of the University of the Ryukyus. The protocol of animal experiments was approved by the Animal Experiment Committee of the University of the Ryukyus (reference number: 5730). Four week-old female BALB/cScl-nu/nu nude mice (Japan SLC, Inc.) were purchased. At 5 weeks of age, they were injected subcutaneously with 1.0 × 10^5^ cells. The mice were euthanized 12 weeks after injection with sodium pentobarbital, and tissue samples were collected for molecular and histological analysis.

### Histopathological analysis

Tumor samples from the nude mice were fixed in 10% phosphate-buffered formalin and embedded in paraffin. The samples were sectioned in 3 μm-thickness and dewaxed with xylene. The sections were examined with conventional hematoxylin and eosin staining. For immunohistochemistry, the sections were heated with 10 mM citrate buffer (pH 6.8) in an electric pot. After antigen retrieval, the sections were treated with 0.3% H_2_O_2,_ and incubated with 1% BSA for 60 min. The sections were then incubated with primary antibodies (shown in Supplemental Table S2) for 60 min followed by HRP-labeled secondary antibody (Nichirei). The signals were visualized using diaminobenzidine (Nichirei).

### Statistical analysis

Data were analyzed by non-repeated measures ANOVA followed by Bonferroni’s multiple comparison test. A *p*-value of < 0.05 was considered as statistically significant.

## Results

### Co-expression of low-risk HPV6 E6 and EBV LMP-1 increased cell proliferation via NF-κB activation and reduced p53 induction

**Fig. 1:**
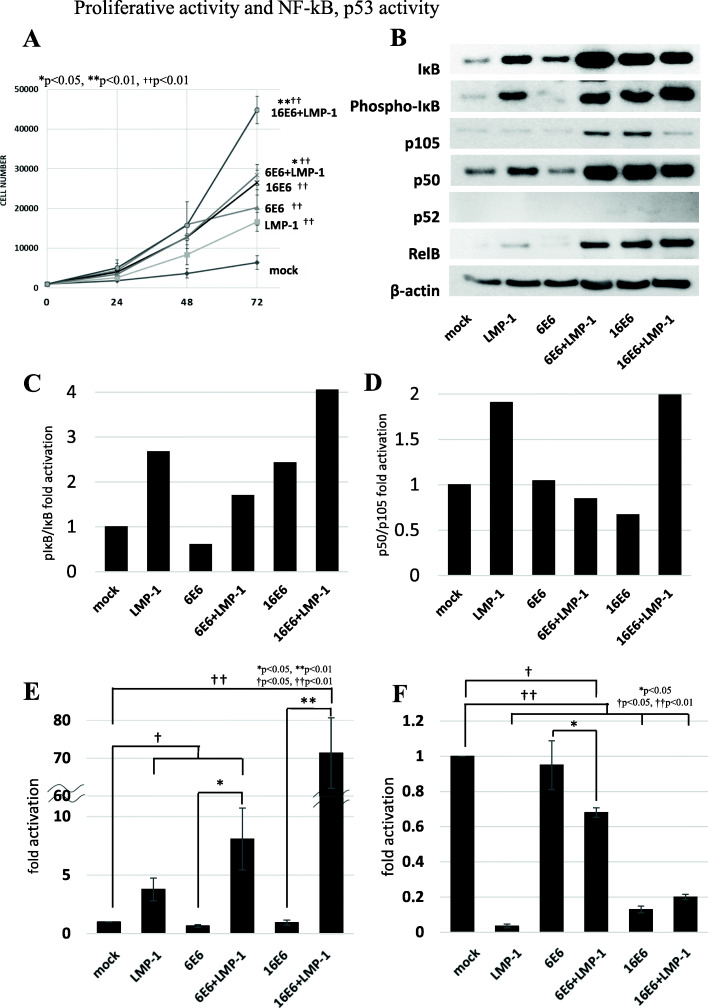
Cell proliferation, NF-κB activity and p53 induction. **A** Cell proliferation was compared among MEFs expressing viral proteins. The cells co-expressing low- or high-risk HPV E6 + EBV LMP-1 (6E6 + LMP-1 and 16E6 + LMP-1) grew faster than those expressing single viral protein and mock cells. Asterisk symbol indicates significant increase in cell number compared with cells expressing HPV E6 alone (*p < 0.05, **p < 0.01). Dagger symbol indicates a significant increase in cell number compared with mock cells (††*p* < 0.01). **B** Low-risk HPV6 E6 + EBV LMP-1 (6E6 + LMP-1) showed phosphorylation of IκB, processing of p105 to p50, and high RelB expression which were comparable to those seen in high-risk HPV16 E6 and EBV LMP-1 (16E6 + LMP-1). **C** and **D**) Relative pIκB/IκB (**C**) and p50/p105 (**D**) ratios of each clone were determined by densitometry. The ratios in mock cells were set to 1.0. Although high-risk HPV16 E6 + EBV LMP-1 (16E6 + LMP-1) showed increased ratios of both pIκB/IκB and p50/p105, low-risk HPV6 E6 + EBV LMP-1 (6E6 + LMP-1) had a high pIκB/IκB ratio but a p105/p50 ratio comparable to mock cells. **E** A luciferase assay for NF-κB activity indicated that low-risk HPV6 E6 + EBV LMP-1 (6E6 + LMP-1) had higher activity than cells expressing HPV6 E6 alone (6E6). However, high-risk HPV16 E6 + EBV LMP-1 (16E6 + LMP-1) showed more than eight-fold increase in activity over low-risk HPV6 E6 + EBV LMP-1 (6E6 + LMP-1). Asterisk and dagger symbols indicate a significant increase of NF-κB activation compared with cells expressing HPV E6 alone (*p < 0.05, **p < 0.01) and mock cells (†p < 0.05, ††p < 0.01), respectively. **F** Induction levels of p53 of each clone were compared through a luciferase assay. p53 expression decreased more in low-risk HPV6 E6 + EBV LMP-1 (6E6 + LMP-1) than those expressing HPV6 E6 alone (6E6). However, p53 suppression in low-risk HPV6 E6 + EBV LMP-1 (6E6 + LMP-1) was lower than that in high-risk HPV16 E6 + EBV LMP-1 (16E6 + LMP-1). Asterisk and dagger symbols indicate a significant decrease of p53 activation compared with cells expressing HPV E6/E7 alone (*p < 0.05) and mock cells (†p < 0.05, ††p < 0.01), respectively

To evaluate the transformation activity by low-risk HPV6 E6 and EBV LMP-1 co-expression, we firstly examined cell proliferation rates using MTT assay. The cells expressing any of the viral proteins grew significantly faster than mock cells (^††^*p* < 0.01) (Fig. 1A). In particular, the cells co-expressing both HPV6 E6 + EBV LMP-1 (6E6 + LMP-1) and HPV16 E6 + EBV LMP-1 (16E6 + LMP-1) showed significantly higher proliferation rates regardless of whether the E6 was from high-risk or low-risk HPV (*p < 0.05, **p < 0.01). HPV6 E7 + EBV LMP-1 (6E7 + LMP-1) also demonstrated elevated proliferation compared with mock cells, as seen in Supplemental Figure S2A.

It has been reported that EBV LMP-1 activates nuclear factor-kappa B (NF-κB) pathways [17], while HPV E6 and E7 suppress or degrade p53 and pRb [1]. To examine the relationship in the cells expressing low-risk HPV6 E6 + EBV LMP-1 (6E6 + LMP-1) between high proliferative activity and NF-κB activity, NF-κB-related proteins and its transactivation activity were measured by Western blotting and luciferase reporter assay, respectively. On Western blotting analyses, low-risk HPV6 E6 + EBV LMP-1 (6E6 + LMP-1) showed phosphorylation of IκB, processing of p105 to p50, and high RelB expression (Fig. 1B) correlated with high pIκB/IκB ratio but comparable p105/p50 one to mock cells (Fig. 1C, D). On reporter assay for NF-κB, low-risk HPV6 E6 + EBV LMP-1 (6E6 + LMP-1) had higher activity than cells expressing only HPV6 E6 (6E6) (*p < 0.05) (Fig. 1E). As EBV LMP-1 suppresses transcription of p53 [18], we performed a reporter assay to examine p53 induction in each cell line. The cells co-expressing low-risk HPV6 E6 + EBV LMP-1 (6E6 + LMP-1) reduced p53 induction significantly more than those expressing HPV6 E6 alone (6E6) (Fig. 1F). However, both NF-κB activation and p53 suppression of low-risk HPV6 E6 + EBV LMP-1 (6E6 + LMP-1) were lower than those resulting from high-risk HPV16 + EBV LMP-1 (16E6 + LMP-1) (Fig. 1E, F). Experiments with HPV11 E6/E7 + EBV LMP-1 (11E6 + LMP-1 and 11E7 + LMP-1) yielded similar findings, as seen in Supplemental Figure. S2B-E.

### Co-expression of low-risk HPV6 E6 and EBV LMP-1 induced DNA damage, escape from apoptosis and suppression of DNA damage response (DDR) under genotoxic condition

**Fig. 2:**
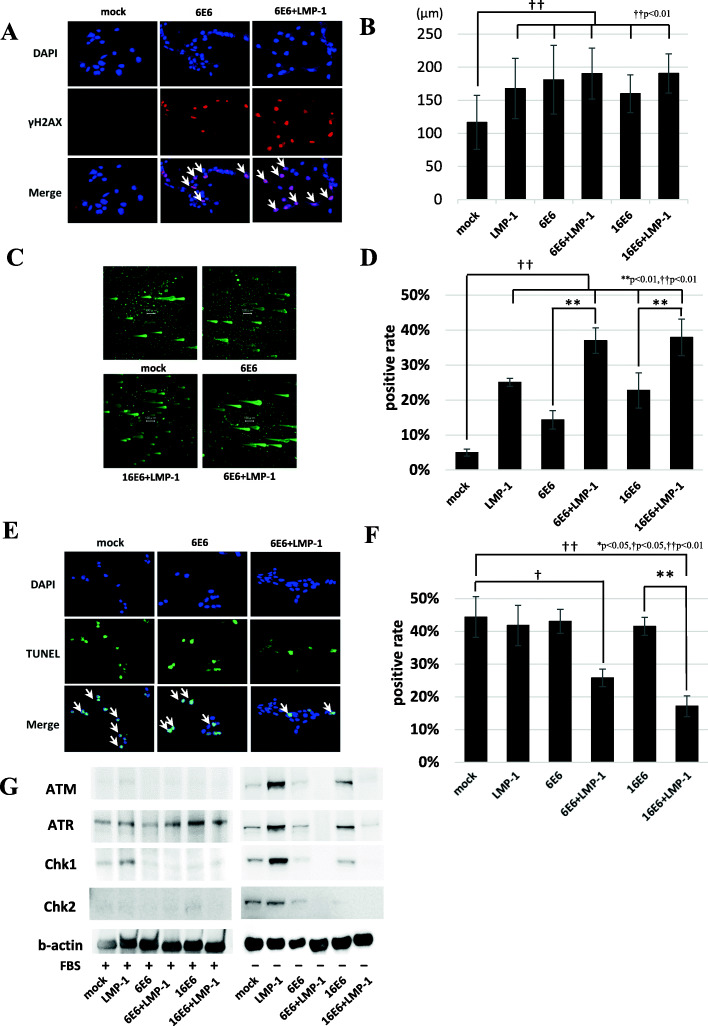
DNA damage, DNA damage response (DDR) and apoptosis. **A** γ-H2AX immunofluorescent staining showed that MEFs expressing viral protein(s) had a higher percentage of γ-H2AX-positive cells than mock cells. However, low-risk HPV6 E6 + EBV LMP-1 (6E6 + LMP-1) induced higher rates of DNA damage than cells expressing HPV6 E6 alone (6E6). Arrows indicate γ-H2AX positive cells. **B** The rates of γ-H2AX positive cells were calculated and compared among the clones. Both low-risk HPV6 E6 + EBV LMP-1 (6E6 + LMP-1) and high-risk HPV16 E6 + EBV LMP-1 (16E6 + LMP-1) showed significantly increased γ-H2AX signals compared with the cells expressing a single viral protein. Asterisk symbol indicates significantly increased rate of γ-H2AX signal (**p < 0.01). **C** A comet assay showed that viral protein expressing cells induced more comet signals than mock cells. More comet signals and longer comet tails were observed in the cells expressing HPV6 E6 + EBV LMP-1 (6E6 + LMP-1). **D** Comet tail length of each cell line was measured using ImageJ software. Comet tail lengths of viral protein expressing cell lines were longer than those of mock cells (††p < 0.01), but there was no significant difference between low-risk HPV6 E6 + EBV LMP-1 (6E6 + LMP-1) and high-risk HPV16 E6 + EBV LMP-1 (16E6 + LMP-1). **E** Low-risk HPV6 E6 + EBV LMP-1 (6E6 + LMP-1) showed less TUNEL signals than mock cells and single low-risk HPV6 E6 (6E6) expressing cells under genotoxic conditions. Arrows indicate TUNEL-positive cells. **F** TUNEL staining rates of each cell line under genotoxic conditions were measured and compared. Asterisk symbol indicates a significant increase in TUNEL-positive cells as detected by immunofluorescent staining (*p < 0.05). **G** Under normal conditions, the ATR-ChK1 pathway was induced across all cell lines. However, ATM-ChK2 proteins were not expressed. Under genotoxic conditions, low-risk HPV6 E6 + EBV LMP-1 (6E6 + LMP-1) and high-risk HPV16 E6 + EBV LMP-1 (16E6 + LMP-1) showed suppression in both the ATR-ChK1 and ATM-ChK2 pathways. FBS: fetal bovine serum

Generally, it is thought that DNA damage occurs in cancer cells by hyperproliferation associated with oncogene signaling. DNA damage activates DNA damage response (DDR) pathway, which transmits signals to various cellular machineries including apoptosis. Therefore, apoptosis is suppressed by derangement of both the apoptotic machinery and DDR [19, 20]. To analyze DNA damage, we performed immunofluorescent staining of γ-H2AX. MEFs expressing viral protein(s) had a higher percentage of γ-H2AX-positive cells than mock cells (Fig. 2A, B and Supplemental Figure S3A, B). In particular, the cells expressing low-risk HPV6/11 E6/E7 + EBV LMP-1 (6E6 + LMP-1, 6E7 + LMP-1, 11E6 + LMP-1 and 11E7 + LMP-1) induced greater DNA damage than the cells expressing low-risk HPV6/11 E6 or E7 alone (6E6, 6E7, 11E6 and 11E7) (Fig. 2A, B and Supplemental Figure S3A, B). A comet assay also showed that low-risk HPV6 E6 + EBV LMP-1 (6E6 + LMP-1) induced longer comet tails than mock cells and increased the comet positivity rate (Fig. 2C, D and Table 1). We performed TUNEL staining under genotoxic conditions to examine resistance to apoptosis. Low-risk HPV6/11 E6/7 + EBV LMP-1 (6E6 + LMP-1, 6E7 + LMP-1, 11E6 + LMP-1 and 11E7 + LMP-1) had a lower percentage of TUNEL-positive cells than MEFs expressing a single viral protein (6E6, 6E7, 11E6 and 11E7) (Fig. 2E, F and Supplemental Figure S3C, D), despite of increased DNA damage (Fig. 2A-D and Supplemental Figure S3A, B). We also assessed DDR by Western blotting. Under normal conditions, the ATR-Chk1 pathway was induced among all the cell lines tested including original CF-1 and mock cells, but ATM-Chk2 proteins were not expressed (Fig. 2G and Supplemental Figure S3E). Under genotoxic conditions, mock cells and the cells expressing a single viral protein induced ATM, ATR, Chk1, and Chk2. However, these DDR proteins were suppressed in cells expressing low-risk HPV6 E6 + EBV LMP-1 (6E6 + LMP-1) suggesting inhibition of DDR (Fig. 2G). Taken together, low-risk HPV6 E6 + EBV LMP-1 (6E6 + LMP-1) caused greater DNA damage and suppression of DDR, leading to mutagenesis identical to that seen with high-risk HPV16 E6 + EBV LMP-1 (16E6 + LMP-1).

**Table 1:** Comet assay

	Comet length (μm ± SD)	Positive rate (%)
**mock**	116.5 ± 40.9	17%
**LMP-1**	167.8 ± 45.3	26%
**6E6**	181.0 ± 51.9	37%
**6E6 + LMP-1**	190.2 ± 38.5	38%
**16E6**	159.9 ± 28.7	35%
**16E6 + LMP-1**	190.4 ± 29.5	41%

### No malignant phenotype was seen in the cells co-expressing low-risk HPV6 E6 and EBV LMP-1

**Fig. 3:**
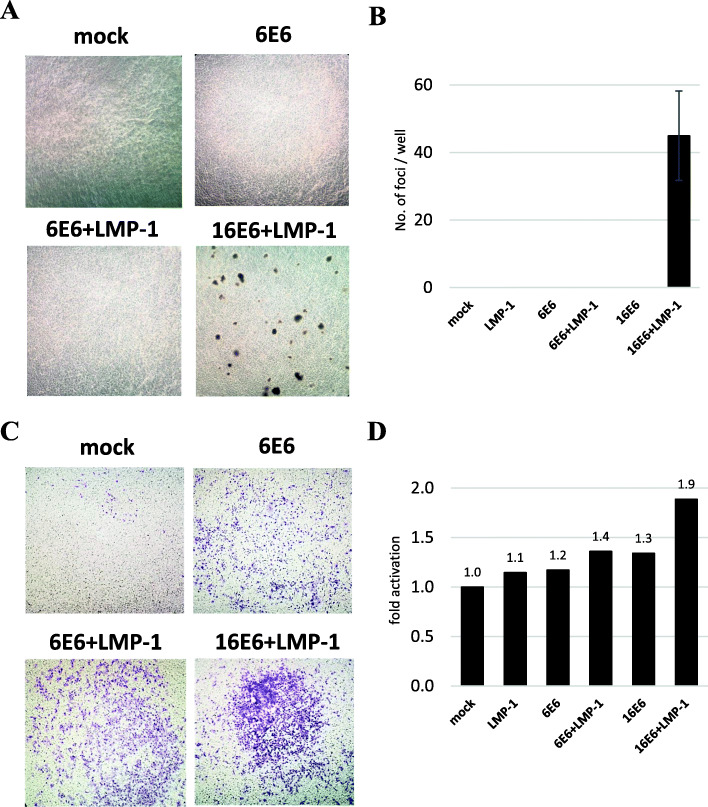
Anchorage-independent growth and invasion. **A** High-risk HPV16 E6 + EBV LMP-1 (16E6 + LMP-1) formed many colonies on DMEM with agar, whereas low-risk HPV6 E6 + EBV LMP-1 (6E6 + LMP-1) was unable to form any colonies. **B** The number of colonies formed on DMEM with agar was counted and compared among clones. High-risk HPV16 E6 + EBV LMP-1 (16E6 + LMP-1) formed about 40 colonies of various sizes, but low-risk HPV6 E6 + EBV LMP-1 (6E6 + LMP-1) demonstrated no colony formation. **C** High-risk HPV16 E6 + EBV LMP-1 (16E6 + LMP-1) displayed higher invasive capacity than other clones, whereas low-risk HPV6 E6 + EBV LMP-1 (6E6 + LMP-1) exhibited only slightly more invasion than mock cells. **D** The invasive capacity of each clone was quantified in relation to the level of mock cells set to 1.0. High-risk HPV16 E6 + EBV LMP-1 (16E6 + LMP-1) showed a two-fold higher invasive capacity than mock cells. However, low-risk HPV6 E6 + EBV LMP-1 (6E6 + LMP-1) demonstrated a slightly increased invasive capacity compared with the single viral protein expressing clones

To assess the malignant features associated with low-risk HPV6 E6 and EBV LMP-1 co-expression, we performed soft agar colony formation and cell invasion assays. In the former, high-risk HPV16 E6 + EBV LMP-1 (16E6 + LMP-1) cells formed abundant colonies of various sizes in the agar medium, but low-risk HPV6 E6 + EBV LMP-1 (6E6 + LMP-1) cells formed no colony (Fig. 3A, B). In cell invasion assay, high-risk HPV16 E6 + EBV LMP-1 (16E6 + LMP-1) cells displayed higher invasive capacity than other clones, while low-risk HPV6/11 E6/E7 + EBV LMP-1 (6E6 + LMP-1, 6E7 + LMP-1, 11E6 + LMP-1 and 11E7 + LMP-1) exhibited only slightly more invasion than mock cells, a result comparable to that with clones expressing only one of the viral proteins (Fig. 3C, D and Supplemental Figure S4A, B). To assess the tumor formation in vivo, we injected the cell lines under the skin of 5 week-old nude mice and observed them for 2 months. Although all the mice injected with high-risk HPV16 E6 + EBV LMP-1 (16E6 + LMP-1) cells developed tumors, no tumor was found in mice injected with low-risk HPV6 E6 + EBV LMP-1 (6E6 + LMP-1) cells, low-risk HPV11 E6/E7 + EBV LMP-1 (11E6 + LMP-1 and 11E7 + LMP-1) cells or mock cells (Table 2 and Supplemental Table S4).

**Table 2:** Tumors in nude mice

	Tumors in nude mice
mock	0/15
6E6 + LMP-1	0/15
16E6 + LMP-1	6/6

### Anchorage-independent growth was suppressed in the cells co-expressing low-risk HPV6 E6 and EBV LMP-1 through downregulation of adhesion signaling

**Fig. 4:**
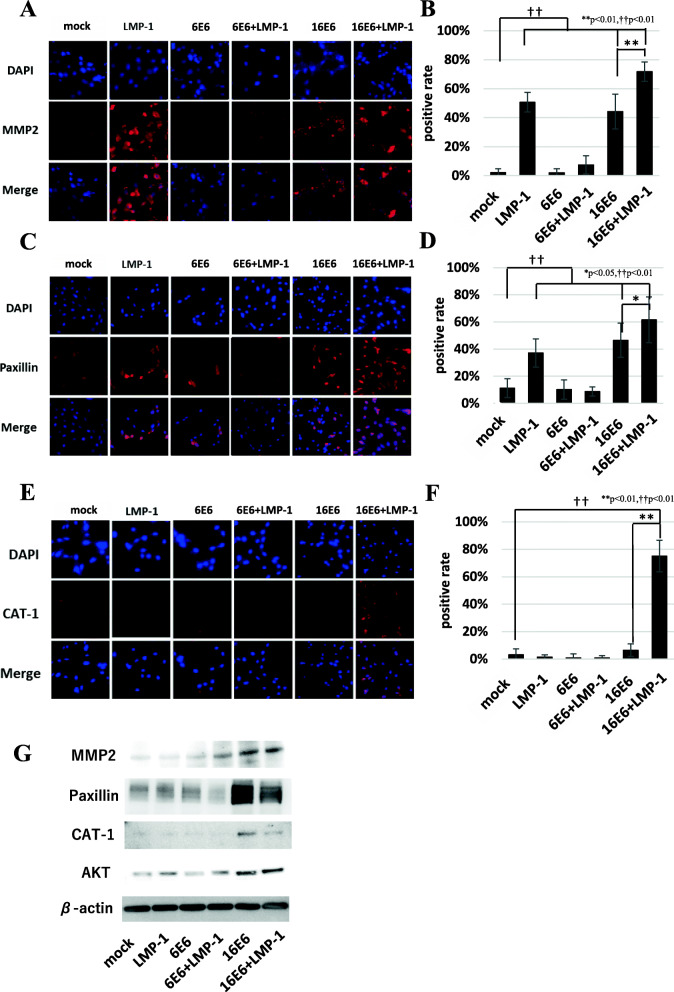
Expression of adhesion molecules. **A** and **B** MMP-2 expression was evaluated by immunofluorescent staining (**A**) and the rate of MMP-2 positive clones was displayed (**B**). Clones only expressing high-risk HPV16 E6 or EBV LMP-1 (LMP-1, 16E6 and 16E6 + LMP-1) showed increased MMP-2 expression compared with low-risk HPV6 E6 + EBV LMP-1 (6E6 + LMP-1). **C** and **D** Immunofluorescent staining of paxillin (**C**) and the relative rate of paxillin-positive clones (**D**) were analyzed. Some of the viral protein expressing clones demonstrated increased levels of paxillin, but low-risk HPV6 E6 + EBV LMP-1 (6E6 + LMP-1) displayed comparable levels with mock cells. **E** and **F** Cat-1 immunofluorescent staining (**E**) and the relative rate of Cat-1 positive clones were evaluated (**F**). Elevated Cat-1 expression was only seen in high-risk HPV16 E6 + EBV LMP-1 (16E6 + LMP-1). Low-risk HPV6 E6 + EBV LMP-1 (6E6 + LMP-1) expressed low levels of Cat-1 that are comparable to those of mock cells. **G** Expression of adhesion molecules was compared among clones. High-risk HPV16 E6 + EBV LMP-1 (16E6 + LMP-1) demonstrated an overall increase in the expression of adhesion molecules and higher Akt expression, whereas low-risk HPV6 E6 + EBV LMP-1 (6E6 + LMP-1) showed low expression and activity

Despite the mutagenetic effects of low-risk HPV6 E6 + EBV LMP-1 (6E6 + LMP-1) cells and low-risk HPV11 E6/E7 + EBV LMP-1 (11E6 + LMP-1 and 11E7 + LMP-1), they were unable to induce transformation (Table 2 and Supplemental Table S4). The most significant difference between low-risk HPV6 E6 + EBV LMP-1 (6E6 + LMP-1) and high-risk HPV16 E6 + EBV LMP-1 (16E6 + LMP-1) cells was invasiveness and anchorage-independent growth. To examine more precisely, we compared the expression level of matrix metalloproteinase-2 (MMP-2), which plays crucial role in cancer invasion. Although high-risk HPV16 E6 alone (16E6) or with EBV LMP-1 (16E6 + LMP-1) was associated with increased MMP-2 expression, low-risk HPV6 E6 (6E6) did not induce MMP-2 expression. Conversely, it suppressed the effect of LMP-1 on MMP-2 induction, which was seen in low-risk HPV6 E6 + EBV LMP-1 (6E6 + LMP-1) (Fig. 4A, B). Anchorage-independent growth is an important neoplastic trait associated with adhesion signaling. It has been reported that control of focal adhesion proteins such as paxillin is important for cancer metastasis, which depends on cellular migration and cell-matrix adhesion [21]. Cool-associated tyrosine-phosphorylated protein-1 (Cat-1) has a crucial role in anchorage-independent growth by interacting with paxillin, which then activates Akt signaling in uterine cervical carcinoma cells [22]. We compared adhesion molecule signaling between low-risk HPV6 E6 + EBV LMP-1 (6E6 + LMP-1) and high-risk HPV16 E6 + EBV LMP-1 (16E6 + LMP-1) using immunocytochemistry and Western blotting. Although some of the viral proteins demonstrated increased levels of paxillin, low-risk HPV6 E6 + EBV LMP-1 (6E6 + LMP-1) displayed comparable level of mock cells (Fig. 4C, D). Elevated Cat-1 expression was only seen in cells with high-risk HPV16 E6 + EBV LMP-1 (16E6 + LMP-1) (Fig. 4E, F). We confirmed similar cytoplasmic expression of MMP-2, paxillin and Cat-1 in the tumors arising in the nude mice injected with high-risk HPV16 E6 + EBV LMP-1 (16E6 + LMP-1) (Supplemental Figure S5). High-risk HPV16 E6 + EBV LMP-1 (16E6 + LMP-1) induced greater Akt signaling, whereas low-risk HPV6 E6 + EBV LMP-1 (6E6 + LMP-1) showed low Akt activity (Fig. 4G). These results suggest that co-infection with a low-risk HPV6 E6 + EBV LMP-1 (6E6 + LMP-1) is incapable of causing invasion and anchorage-independent growth because such an expression would not affect adhesion signaling by MMP-2, paxillin, and Cat-1 in the same way that high-risk HPV16 E6 and EBV LMP-1 co-expression does.

Collectively, these data indicate that low-risk HPV and EBV co-infections cannot induce malignant transformation because they do not disrupt cell adhesion signaling, but on the other hand, they do induce preneoplastic lesions that have substantial DNA damage and suppressed DDR.

## Discussion

Our study demonstrated that co-expression of low-risk HPV6/11 E6/E7 and EBV LMP-1 (HPV6 E6 + LMP-1, HPV6 E7 + LMP-1, HPV11 E6 + LMP-1 and HPV11 E7 + LMP-1) led to increased cell proliferation, elevated NF-κB activity, and reduced induction of p53. These co-expressing clones also induced DNA damage, which did not evoke apoptosis and DDR, implying that the accumulated DNA damage triggers mutation. However, in contrast to finding with high-risk HPV16 E6 + EBV LMP-1 (16E6 + LMP-1), low-risk HPV6 E6 + EBV LMP-1 (6E6 + LMP-1) did not induce anchorage-independent growth, invasiveness and tumor formation in nude mice. These findings suggest that a co-infection with a low-risk HPV and EBV increases mutagenicity but does not cause malignant transformation.

Compared with high-risk HPV E6 and E7, low-risk HPV genes had little effect on p53 and pRb, eliciting weak degradation of those tumor suppressor genes [1]. This may explain why low-risk HPVs evoke neither immortalization nor transformation.

In previous reports, we demonstrated that dual expression of high-risk HPV16 E6 and EBV LMP-1 in primary MEFs induced transformation, whereas either viral protein alone did not [15]. In addition, transformation of cells co-expressing high-risk HPV16 E6 and EBV LMP-1 is associated with suppression of DDR and increased NF-κB activity [15]. In a study on LMP-1 expressing B-lymphoma line, DDR was blocked by suppression of the DNA damage sensor, ATM and its downstream protein Chk2 [23]. LMP-1 is also known to induce immortalization of primary human lymphocytes by suppression of apoptosis through NF-κB activation [24]. In addition, LMP-1 has been reported to induce transformation of immortalized human keratinocytes and confer invasion capacity through suppression of E-cadherin expression [25, 26].

In the present study, low-risk HPV6/11 E6/E7 + EBV LMP-1 (6E6 + LMP-1, 11E6 + LMP-1 and 11E7 + LMP-1) activated the NF-κB pathway and suppressed p53 transcriptional activity, an effect not seen in cells expressing low-risk HPV6/11 E6 or E7 alone (6E6, 11E6 and 11E7). It has been reported that LMP-1 activates NF-κB and suppresses transcription of p53, leading to genome instability and inhibition of apoptosis [18, 27]. However, we found that low-risk HPV6 E6 and EBV LMP-1 co-expression was unable to cause transformation. These findings indicate that increased NF-κB activity and suppression of DDR, while leading to mutagenesis, are insufficient for low-risk HPV to induce carcinogenesis.

One striking difference between co-expression of EBV LMP-1 with E6 or E7 from low- versus high-risk HPV was the effects on anchorage-independent growth. Scaffold proteins such as paxillin play an important role in regulating cell migration. Paxillin links the cell membrane to the actin cytoskeleton, and recruits tyrosine kinases such as FAK and Src [21]. Tong and Howley reported that paxillin interacts with high-risk HPV E6 to cause transformation, but these effects are not seen with low-risk HPV E6 [28]. Another scaffold protein, Cat-1 interacts with paxillin and promotes anchorage-independent growth by Akt activation in HeLa cells [22]. Our data showed that low-risk HPV6 E6 + EBV LMP-1 (6E6 + LMP-1) induced less expression of paxillin and Cat-1 than high-risk HPV16 E6 + EBV LMP-1 (16E6 + LMP-1). Probably, the insufficient anchorage-independent growth with low-risk HPV6 E6 + EBV LMP-1 (6E6 + LMP-1) may be explained by downregulation of cell signaling via paxillin and Cat-1. MMP-2 is associated with metastasis of oral squamous cell carcinoma cells [29]. Zhu et al. reported that HPV16 E6 and E7 proteins upregulate MMP-2 and MMP-9, and promote cell migration of cervical cancer cells [30]. Similar to high-risk HPV E6 and E7, EBV LMP-1 protein isolated from nasopharyngeal carcinoma also induces MMPs [31]. As with our other findings, MMP-2 expression with low-risk HPV6 E6 + EBV LMP-1 (6E6 + LMP-1) was lower than with high-risk HPV16 E6 + EBV LMP-1 (16E6 + LMP-1). Because MMPs are induced via the NF-κB pathway [32, 33], our data suggest that differences in NF-κB activity in low- versus high-risk HPV co-infection with EBV are associated with differences in anchorage-independent growth.

As summarized in Table 3, our findings with regard to the ability of HPV to affect various factors involved oncogenesis indicate that low-risk HPV proteins alone cause little effect but that low-risk HPV E6 or E7 along with EBV LMP-1 leads to significant mutagenesis without malignant transformation seen with high-risk HPV16 E6 + EBV LMP-1 (16E6 + LMP-1). Several investigators have reported that malignant tumors can develop from benign low-risk HPV associated lesions by smoking or irradiation [34–36]. Because low-risk HPV E6/E7 + EBV LMP-1 (6E6 + LMP-1, 6E7 + LMP-1, 11E6 + LMP-1 and 11E7 + LMP-1) induces more mutagenesis than infection with low-risk HPV E6/E7 alone (6E6, 6E7, 11E6 and 11E7), co-infection of low-risk HPV and EBV may therefore induce precancerous lesions that could be more easily transformed if they are subsequently subjected to further mutagenic effects.

**Table 3:** Summary

	Low-risk HPV	Low-risk HPV + EBV	High-risk HPV + EBV
**Proliferative activity**	–	++	+++
**DNA damage**	+	+++	+++
**Apoptotic suppression**	–	+	++
**NF-κB activation**	–	++	+++
**Inactivation of p53**	–	++	+++
**Invasive ability**	–	±	+
**Anchorage-independent growth**	–	–	+
**Tumor formation in nude mice**	–	–	+
**Activation of adhesion signaling including paxillin, MMP2**	–	–	+

## Conclusions

In summary, our research demonstrated that the co-expression of low-risk HPV6/11 E6/E7 and EBV LMP-1 does not induce malignant transformation, but it allows accumulation of somatic mutations secondary to increased DNA damage and suppression of DDR. Thus, double infection of low-risk HPV and EBV could lead to precancerous lesions.

## Supplementary Information

**Additional file 1:**

**Additional file 2: Figure S1.** Viral gene(s) expression of each clones.

**Additional file 3: Table S1.** Primers used for RT-PCR. **Table S2.** Primary antibodies used for immunocytochemistry and immunohistochemistry.

**Additional file 4: Table S3.** Primary antibodies used for Western blotting. **Table S4.** Tumors in nude mice.

**Additional file 5: Figure S2.** Cell proliferation, NF-κBactivity and p53 induction.

**Additional file 6: Figure S3.** DNA damage, DNA damage response (DDR) and apoptosis.

**Additional file 7: Figure S4.** Invasive capacity.

**Additional file 8: Figure S5.** Expression of adhesion molecules in tumors arising from nude mice.

**Additional file 9: Figure S6.** Original blots shown in Fig. 1B.

**Additional file 10: Figure S7A.** Original blots shown in Fig. 2G. **Figure S7B.** Original blots shown in Fig. 2G.

**Additional file 11: Figure S8.** Original blots shown in Fig. 4G.

**Additional file 12: Figure S9A.** Original blots shown in Supplemental Figure S2E. **Figure S9B.** Original blots shown in Supplemental Fig. S2E.

**Additional file 13: Figure S10.** Original blots shown in Supplemental Figure S3E.

## Data Availability

The datasets used and/or analyzed during the current study are available from the corresponding author on reasonable request.

## Abbreviations

HPV: Human papillomavirus
EBV: Epstein-Barr virus
LMP-1: Latent membrane protein-1
DDR: DNA damage response
MEF: Mouse embryonic fibroblast
MTT: Methyl thiazolyl tetrazolium, 3-(4,5-Dimethyl-2-thiazolyl)-2,5-diphenyl-2H-tetrazolium bromide
TUNEL: TdT-mediated dUTP-biotin nick end labeling
MMP: Matrix metalloproteinase
Cat-1: Cool-associated tyrosine-phosphorylated protein-1

## References

[1] Doorbar J, Quint W, Banks L, Bravo IG, Stoler M, Broker TR, Stanley MA (2012). The biology and life-cycle of human papillomaviruses. Vaccine.

[2] Munoz N, Bosch FX, de Sanjose S, Herrero R, Castellsague X, Shah KV, Snijders PJ, Meijer CJ, International Agency for Research on Cancer multicenter cervical Cancer study G (2003). Epidemiologic classification of human papillomavirus types associated with cervical cancer. N Engl J Med.

[3] Li X, Coffino P (1996). High-risk human papillomavirus E6 protein has two distinct binding sites within p53, of which only one determines degradation. J Virol.

[4] Munger K, Werness BA, Dyson N, Phelps WC, Harlow E, Howley PM (1989). Complex formation of human papillomavirus E7 proteins with the retinoblastoma tumor suppressor gene product. EMBO J.

[5] Lee LA, Huang CG, Tsao KC, Liao CT, Kang CJ, Chang KP, Huang SF, Chen IH, Fang TJ, Li HY, Yang SL, Lee LY, Hsueh C, Chen TC, Lin CY, Fan KH, Wang HM, Ng SH, Chang YL, Lai CH, Shih SR, Yen TC (2013). Increasing rates of low-risk human papillomavirus infections in patients with oral cavity squamous cell carcinoma: association with clinical outcomes. J Clin Virol.

[6] Kreimer AR, Clifford GM, Boyle P, Franceschi S (2005). Human papillomavirus types in head and neck squamous cell carcinomas worldwide: a systematic review. Cancer Epidemiol Biomark Prev.

[7] Jiang R, Ekshyyan O, Moore-Medlin T, Rong X, Nathan S, Gu X, Abreo F, Rosenthal EL, Shi M, Guidry JT, Scott RS, Hutt-Fletcher LM, Nathan CAO (2015). Association between human papilloma virus/Epstein-Barr virus coinfection and oral carcinogenesis. J Oral Pathol Med.

[8] Polz-Gruszka D, Morshed K, Stec A, Polz-Dacewicz M (2015). Prevalence of human papillomavirus (HPV) and Epstein-Barr virus (EBV) in oral and oropharyngeal squamous cell carcinoma in South-Eastern Poland. Infect Agent Cancer.

[9] Tsuhako K, Nakazato I, Miyagi J, Iwamasa T, Arasaki A, Hiratsuka H, Sunakawa H, Kohama G, Abo T (2000). Comparative study of oral squamous cell carcinoma in Okinawa, southern Japan and Sapporo in Hokkaido, northern Japan; with special reference to human papillomavirus and Epstein-Barr virus infection. J Oral Pathol Med.

[10] Hutt-Fletcher LM (2007). Epstein-Barr virus entry. J Virol.

[11] Young LS, Murray PG (2003). Epstein-Barr virus and oncogenesis: from latent genes to tumours. Oncogene.

[12] Roberts ML, Cooper NR (1998). Activation of a ras-MAPK-dependent pathway by Epstein-Barr virus latent membrane protein 1 is essential for cellular transformation. Virology.

[13] Kulwichit W, Edwards RH, Davenport EM, Baskar JF, Godfrey V, Raab-Traub N (1998). Expression of the Epstein-Barr virus latent membrane protein 1 induces B cell lymphoma in transgenic mice. Proc Natl Acad Sci U S A.

[14] Lu JJ, Chen JY, Hsu TY, Yu WC, Su IJ, Yang CS (1997). Cooperative interaction between Bcl-2 and Epstein-Barr virus latent membrane protein 1 in the growth transformation of human epithelial cells. J Gen Virol.

[15] Shimabuku T, Tamanaha A, Kitamura B, Tanabe Y, Tawata N, Ikehara F, Arakaki K, Kinjo T (2014). Dual expression of Epstein-Barr virus, latent membrane protein-1 and human papillomavirus-16 E6 transform primary mouse embryonic fibroblasts through NF-kappaB signaling. Int J Clin Exp Pathol.

[16] Tamanaha-Nakasone A, Uehara K, Tanabe Y, Ishikawa H, Yamakawa N, Toyoda Z, Kurima K, Kina S, Tsuneki M, Okubo Y, Yamaguchi S, Utsumi D, Takahashi K, Arakawa H, Arasaki A, Kinjo T (2019). K1 gene transformation activities in AIDS-related and classic type Kaposi's sarcoma: correlation with clinical presentation. Sci Rep.

[17] Gires O, Zimber-Strobl U, Gonnella R, Ueffing M, Marschall G, Zeidler R, Pich D, Hammerschmidt W (1997). Latent membrane protein 1 of Epstein-Barr virus mimics a constitutively active receptor molecule. EMBO J.

[18] Liu MT, Chang YT, Chen SC, Chuang YC, Chen YR, Lin CS, Chen JY (2005). Epstein-Barr virus latent membrane protein 1 represses p53-mediated DNA repair and transcriptional activity. Oncogene.

[19] Hanahan D, Weinberg RA (2011). Hallmarks of cancer: the next generation. Cell.

[20] Kuo LJ, Yang LX (2008). Gamma-H2AX - a novel biomarker for DNA double-strand breaks. In Vivo.

[21] Turner CE (2000). Paxillin and focal adhesion signalling. Nat Cell Biol.

[22] Yoo SM, Latifkar A, Cerione RA, Antonyak MA (2017). Cool-associated tyrosine-phosphorylated protein 1 is required for the Anchorage-independent growth of cervical carcinoma cells by binding Paxillin and promoting AKT activation. J Biol Chem.

[23] Gruhne B, Sompallae R, Masucci MG (2009). Three Epstein-Barr virus latency proteins independently promote genomic instability by inducing DNA damage, inhibiting DNA repair and inactivating cell cycle checkpoints. Oncogene.

[24] Devergne O, Hatzivassiliou E, Izumi KM, Kaye KM, Kleijnen MF, Kieff E, Mosialos G (1996). Association of TRAF1, TRAF2, and TRAF3 with an Epstein-Barr virus LMP1 domain important for B-lymphocyte transformation: role in NF-kappaB activation. Mol Cell Biol.

[25] Hu LF, Chen F, Zheng X, Ernberg I, Cao SL, Christensson B, Klein G, Winberg G (1993). Clonability and tumorigenicity of human epithelial cells expressing the EBV encoded membrane protein LMP1. Oncogene.

[26] Fahraeus R, Chen W, Trivedi P, Klein G, Obrink B (1992). Decreased expression of E-cadherin and increased invasive capacity in EBV-LMP-transfected human epithelial and murine adenocarcinoma cells. Int J Cancer.

[27] Husaini R, Ahmad M, Soo-Beng Khoo A (2011). Epstein-Barr virus latent membrane protein LMP1 reduces p53 protein levels independent of the PI3K-Akt pathway. BMC Res Notes.

[28] Tong X, Howley PM (1997). The bovine papillomavirus E6 oncoprotein interacts with paxillin and disrupts the actin cytoskeleton. Proc Natl Acad Sci U S A.

[29] Kawamata H, Nakashiro K, Uchida D, Harada K, Yoshida H, Sato M (1997). Possible contribution of active MMP2 to lymph-node metastasis and secreted cathepsin L to bone invasion of newly established human oral-squamous-cancer cell lines. Int J Cancer.

[30] Zhu D, Ye M, Zhang W (2015). E6/E7 oncoproteins of high risk HPV-16 upregulate MT1-MMP, MMP-2 and MMP-9 and promote the migration of cervical cancer cells. Int J Clin Exp Pathol.

[31] Lee DC, Chua DT, Wei WI, Sham JS, Lau AS (2007). Induction of matrix metalloproteinases by Epstein-Barr virus latent membrane protein 1 isolated from nasopharyngeal carcinoma. Biomed Pharmacother.

[32] Li J, Lau GK, Chen L, Dong SS, Lan HY, Huang XR, Li Y, Luk JM, Yuan YF, Guan XY (2011). Interleukin 17A promotes hepatocellular carcinoma metastasis via NF-kB induced matrix metalloproteinases 2 and 9 expression. PLoS One.

[33] Bond M, Fabunmi RP, Baker AH, Newby AC (1998). Synergistic upregulation of metalloproteinase-9 by growth factors and inflammatory cytokines: an absolute requirement for transcription factor NF-kappa B. FEBS Lett.

[34] Lindeberg H, Syrjanen S, Karja J, Syrjanen K (1989). Human papillomavirus type 11 DNA in squamous cell carcinomas and pre-existing multiple laryngeal papillomas. Acta Otolaryngol.

[35] Kashima H, Wu TC, Mounts P, Heffner D, Cachay A, Hyams V (1988). Carcinoma ex-papilloma: histologic and virologic studies in whole-organ sections of the larynx. Laryngoscope.

[36] Lindeberg H, Elbrond O (1991). Malignant tumours in patients with a history of multiple laryngeal papillomas: the significance of irradiation. Clin Otolaryngol Allied Sci.

