# Supplementary material for: Co-expression of low-risk HPV E6/E7 and EBV LMP-1 leads to precancerous lesions by DNA damage
Source: BMC Cancer. 2021 Jun 10;21:688. doi: 10.1186/s12885-021-08397-0 (PMC8194219; doi:10.1186/s12885-021-08397-0)
Supplement: Supplementary file 1 — Additional file 1: [file 12885_2021_8397_MOESM1_ESM.pdf]

Supplementary Information

Co-expression of low-risk HPV E6/E7 and EBV LMP-1 leads to precancerous lesions by DNA damage

Karina Uehara [1,4], Yasuka Tanabe [1], Shintaro Hirota [1], Saki Higa [1], Zensei Toyoda [1], Kiyoto Kurima [2], Shinichiro Kina [3], Toshiyuki Nakasone [4], Akira Arasaki [4], Takao Kinjo [1*]

[1] Division of Morphological Pathology, Department of Basic Laboratory Sciences, School of Health Sciences, University of the Ryukyus, 207 Uehara, Nishihara, Okinawa, 903-0215, Japan.

[2] Neurobiology Research Unit, Okinawa Institute of Science and Technology Graduate University, 1919-1 Tancha, Onna, Okinawa, 904-0412, Japan.

[3] Molecular Pharmacology and Oncology, Gunma University, Maebashi, Gunnma 371-8511, Japan

[4] Department of Oral and Maxillofacial Functional Rehabilitation, Graduate School of Medicine, University of the Ryukyus, 207 Uehara, Nishihara, Okinawa, 903-0215, Japan.

Correspondence: Takao Kinjo, MD, PhD

Division of Morphological Pathology, Department of Basic Laboratory Sciences, School of Health Sciences,

University of the Ryukyus, 207 Uehara, Nishihara, Okinawa 903-0215, Japan.

Tel: +81-98-895-1278, Fax: +81-98-895-1434, E-mail: kinjotko@med.u-ryukyu.ac.jp

Supplemental Methods

Viral gene transduction

The plasmids pMSCV-*puro*-*6E6* and pMSCV-*puro*-*6E7* were transfected into CF-1 with or without pMSCV-*neo*-*LMP-1* co-transfection using Lipofectamine 2000 (Thermo Fisher Scientific) and incubated for 48 h to construct CF-1 6E6, CF-1 6E6+LMP-1, CF-1 6E7, CF-1 6E7+LMP-1 and CF-1 6E6+6E7. The results of transfection experiments were shown in Supplemental Figures S2A, S3B, S3D and S4B.

Supplemental Figure S1 Viral gene(s) expression of each clone

All clones expressed *G3PDH* and target viral gene(s). Although two RT-PCR products were observed in *HPV6 E6* and *HPV11 E6*, a DNA sequence analysis confirmed genuine viral gene expression for both. RT (+) indicates that cDNA was generated after reverse-transcription, whereas RT (-) shows the total RNA before reverse-transcription.

Supplemental Figure S2 Cell proliferation, NF-κB activity and p53 induction

(A) Low-risk HPV6 E6/E7 + EBV LMP-1 (6E6+LMP-1 and 6E7+LMP-1) expressed more increased PCNA signals than single viral gene expressing clones suggesting elevated cell proliferation.

(B) Low-risk HPV11 E6/E7 + EBV LMP-1 (11E6+LMP-1 and 11E7+LMP-1) showed higher proliferation than cells expressing a single viral protein.

(C) The NF-κB activity was increased in low-risk HPV11 E6/E7 + EBV LMP-1 (11E6+LMP-1 and 11E7+LMP-1).

(D) Low-risk HPV11 E6/E7 + EBV LMP-1 (11E6+LMP-1 and 11E7+LMP-1) demonstrated decrease in p53 induction.

(E) Low-risk HPV6/11 E6/E7 alone (6E6, 11E6 and 11E7) expressed p53 protein, whereas low-risk HPV6/11 E6/E7 + EBV LMP-1 (6E6+LMP-1, 11E6+LMP-1 and 11E7+LMP-1) showed a decreased

level of p53. Notably, co-expression of HPV11 E6 or E7 with EBV LMP-1 (11E6+LMP-1 and 11E7+LMP-1) suppressed p53 phosphorylation. Low-risk HPV6/11 E6/E7 + EBV LMP-1 (6E6+LMP-1, 11E6+LMP-1 and 11E7+LMP-1) expressed less pRb compared with the cells expressing only the low-risk HPV6/11 E6/E7 (6E6, 11E6 and 11E7).

Supplemental Figure S3 DNA damage, DNA damage response (DDR) and apoptosis

(A) Low-risk HPV11 E6/E7 + EBV LMP-1 (11E6+LMP-1 and 11E7+LMP-1) induced more DNA damage than low-risk HPV11 E6 or E7 alone (11E6 and 11E7).

(B) Viral gene expressing clones induced DNA damage compared with mock.

(C) Under genotoxic conditions, apoptosis was suppressed in low-risk HPV11 E6/E7 + EBV LMP-1 (11E6+LMP-1 and 11E7+LMP-1).

(D) Low-risk HPV6 E6/E7 + EBV LMP-1 (6E6+LMP-1 and 6E7+LMP-1) showed resistance to apoptosis compared with single viral gene expressing clones (6E6 and 6E7).

(E) Regardless of geneticin selection, the ATR-Chk1 pathway is induced in CF-1 and mock cells (CF-1 neo). With the selection, ATM protein abundance is slightly increased in CF-1 neo.

Supplemental Figure S4 Invasive capacity

(A) The invasive capacity of low-risk HPV11 E6/E7 + EBV LMP-1 (11E6+LMP-1 and 11E7+LMP-1) was slightly increased compared with low-risk HPV11 E6 or E7 alone (11E6 and 11E7).

(B) The invasive capacity of low-risk HPV6 E6/E7 + EBV LMP-1 (6E6+LMP-1 and 6E7+LMP-1) was slightly increased compared with low-risk HPV6 E6 or E7 alone (6E6 and 6E7).

Supplemental Figure S5 Expression of adhesion molecules in tumors arising from nude mice

Cytoplasmic expression of adhesion molecules such as MMP-2, paxillin and Cat-1 was seen in tumors from nude mice injected with high-risk HPV16 E6 + EBV LMP-1 (16E6+LMP-1).

Supplemental Figure S6 Original blots shown in Figure 1B

Supplemental Figure S7A Original blots shown in Figure 2G

Supplemental Figure S7B Original blots shown in Figure 2G

Supplemental Figure S8 Original blots shown in Figure 4G

Supplemental Figure S9A Original blots shown in Supplemental Figure S2E

Supplemental Figure S9B Original blots shown in Supplemental Figure S2E

Supplemental Figure S10 Original blots shown in Supplemental Figure 3E
