# Supplementary figures and images for: Co-expression of low-risk HPV E6/E7 and EBV LMP-1 leads to precancerous lesions by DNA damage
Source: BMC Cancer. 2021 Jun 10;21:688. doi: 10.1186/s12885-021-08397-0 (PMC8194219; doi:10.1186/s12885-021-08397-0)

**Supplemental Figure S1** Viral gene(s) expression of each clones

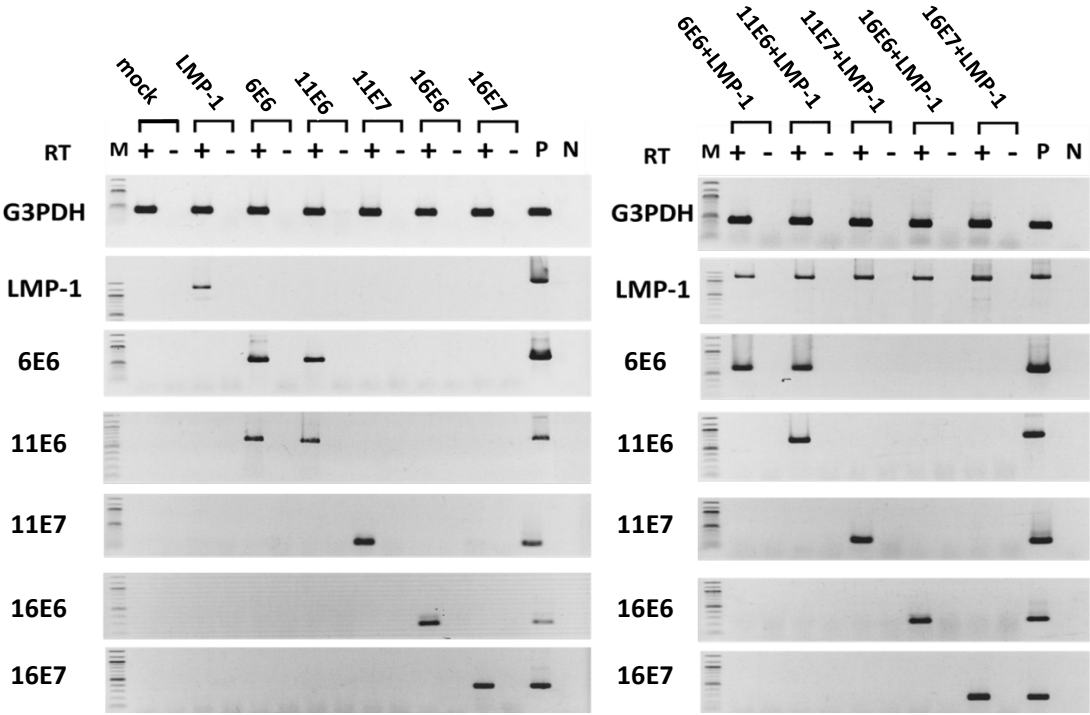

Supplement: Supplementary file 2 — Additional file 2: Figure S1. Viral gene(s) expression of each clones. [file 12885_2021_8397_MOESM2_ESM.pdf]

Supplemental Figure S2 Cell proliferation, NF-κB activity and p53 induction

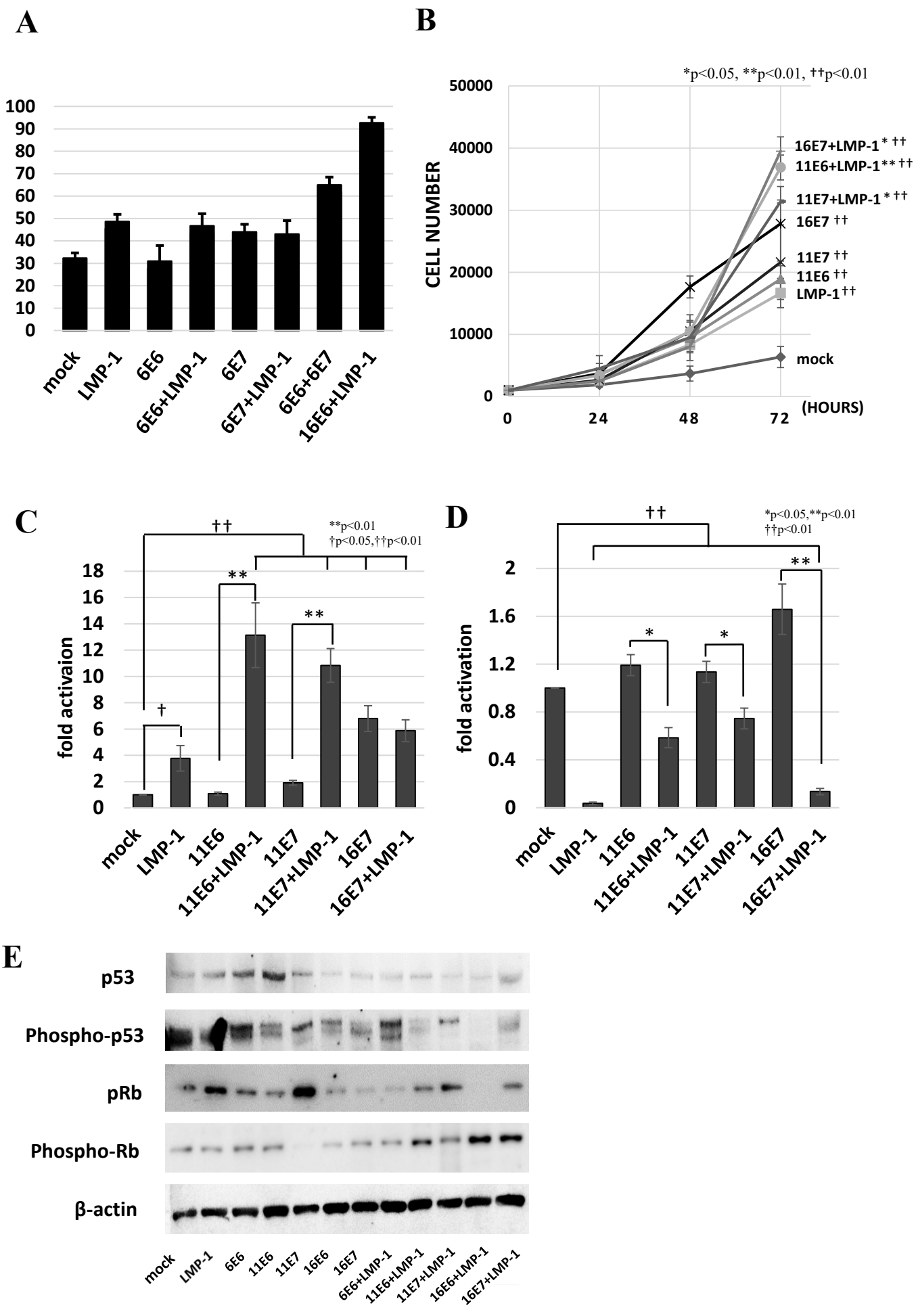

Supplement: Supplementary file 5 — Additional file 5: Figure S2. Cell proliferation, NF-κBactivity and p53 induction. [file 12885_2021_8397_MOESM5_ESM.pdf]

Supplemental Figure S3 DNA damage, DNA damage response (DDR) and apoptosis

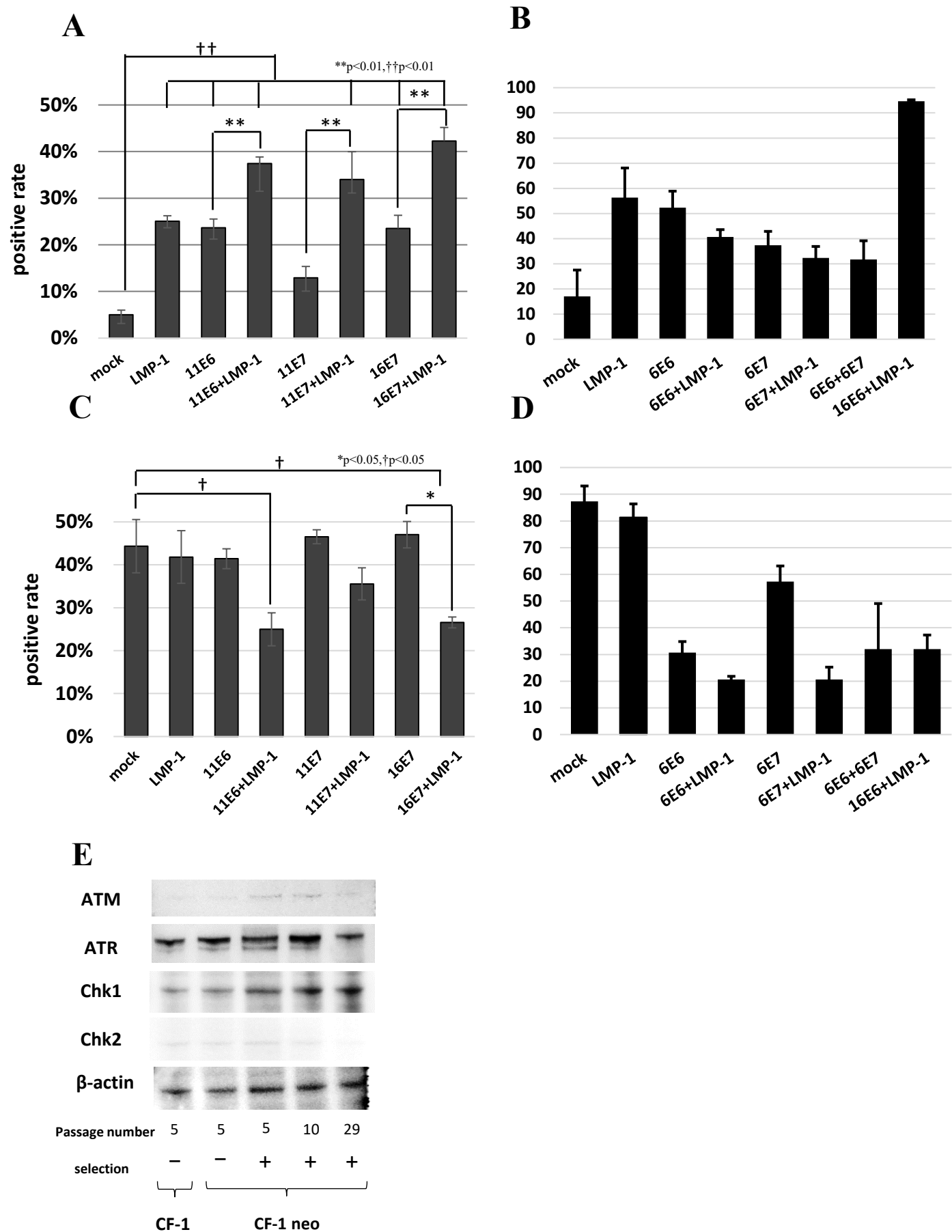

Supplement: Supplementary file 6 — Additional file 6: Figure S3. DNA damage, DNA damage response (DDR) and apoptosis. [file 12885_2021_8397_MOESM6_ESM.pdf]

Supplemental Figure S4 Invasive capacity

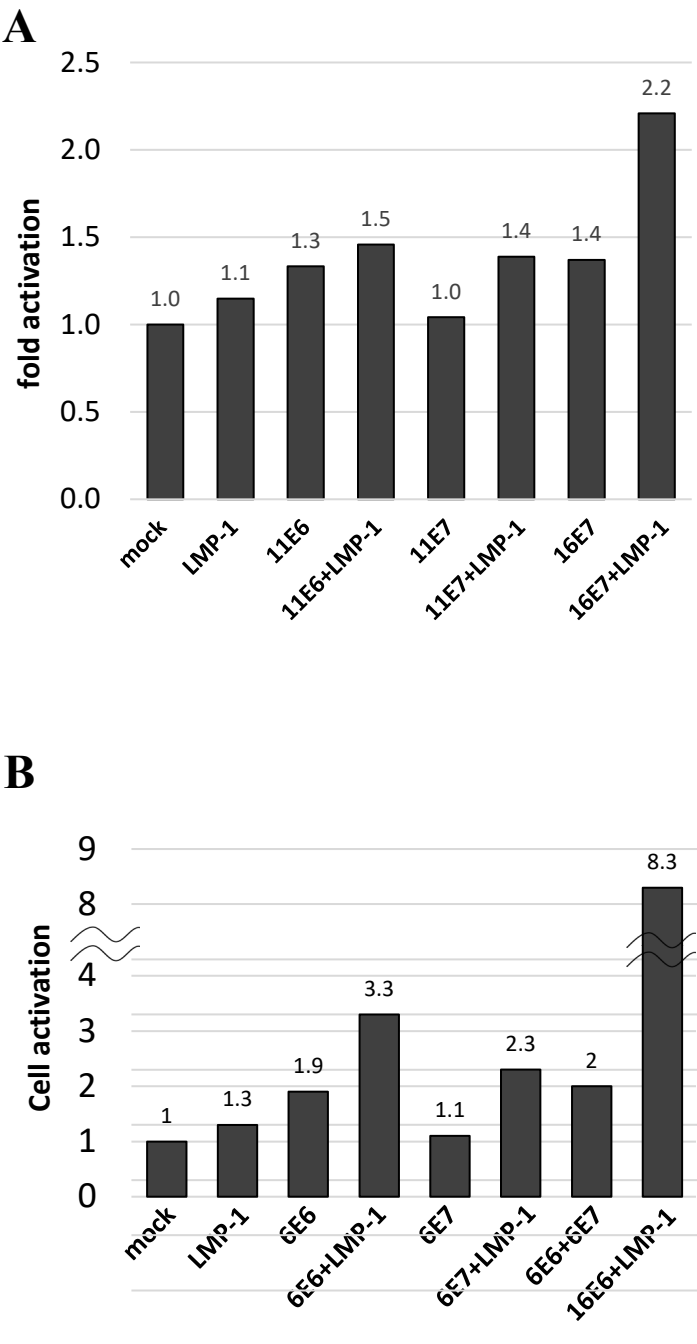

Supplement: Supplementary file 7 — Additional file 7: Figure S4. Invasive capacity. [file 12885_2021_8397_MOESM7_ESM.pdf]

**Supplemental Figure S5** Expression of adhesion molecules in tumors arising from nude mice

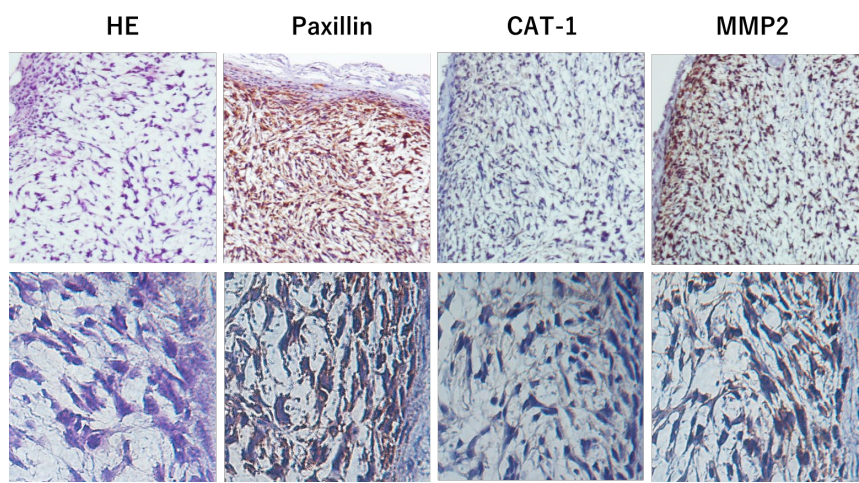

Supplement: Supplementary file 8 — Additional file 8: Figure S5. Expression of adhesion molecules in tumors arising from nude mice. [file 12885_2021_8397_MOESM8_ESM.pdf]

Supplemental Figure S6: Original blots shown in Figure 1B

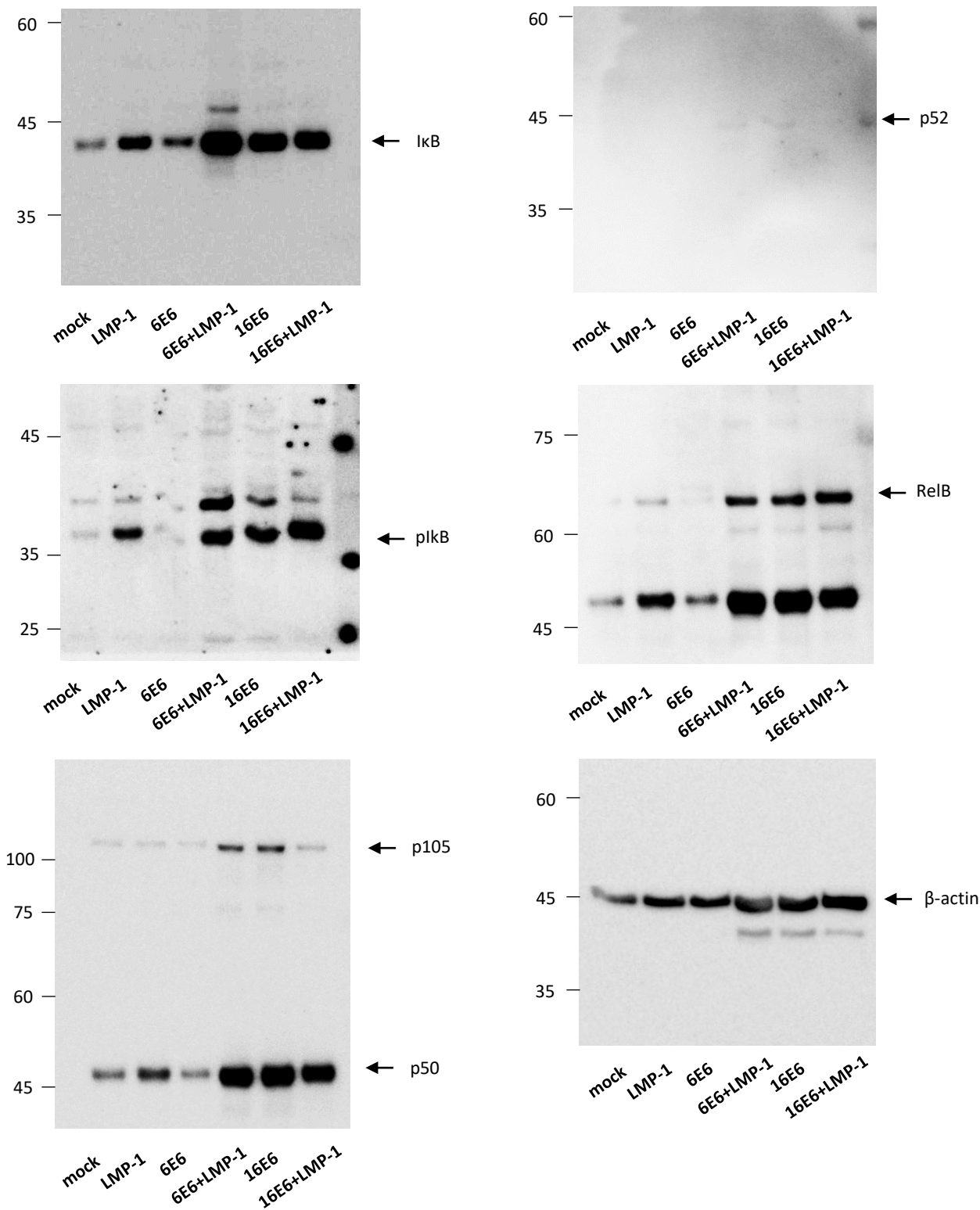

Supplement: Supplementary file 9 — Additional file 9: Figure S6. Original blots shown in Fig. 1B. [file 12885_2021_8397_MOESM9_ESM.pdf]

Supplemental Figure S8: Original blots shown in Figure 4G

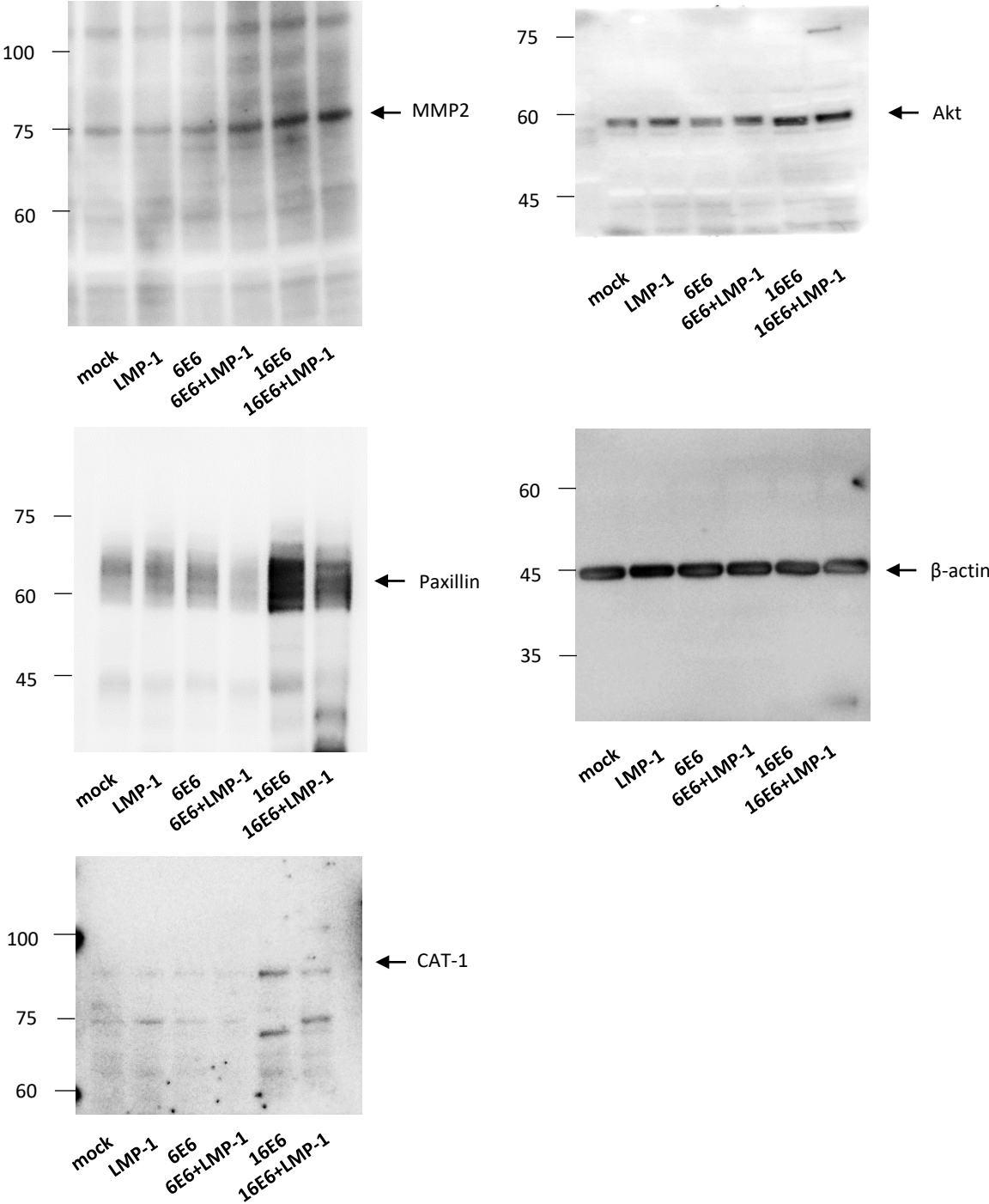

Supplement: Supplementary file 11 — Additional file 11: Figure S8. Original blots shown in Fig. 4G. [file 12885_2021_8397_MOESM11_ESM.pdf]

**Supplemental Figure S10:** Original blots shown in Supplemental Figure S3E

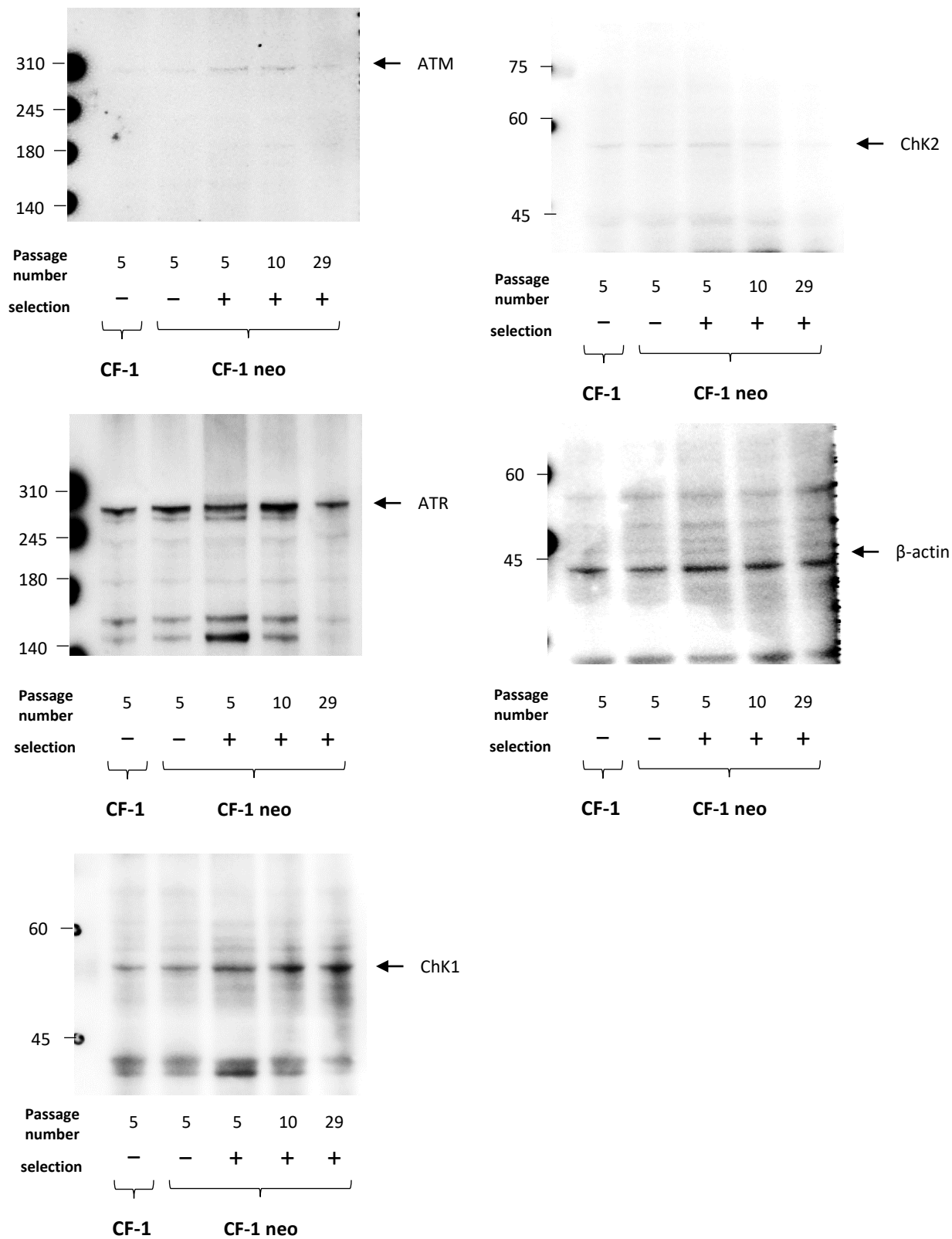

Supplement: Supplementary file 13 — Additional file 13: Figure S10. Original blots shown in Supplemental Figure S3E. [file 12885_2021_8397_MOESM13_ESM.pdf]
