# Supplementary material for: Co-expression of low-risk HPV E6/E7 and EBV LMP-1 leads to precancerous lesions by DNA damage
Source: BMC Cancer. 2021 Jun 10;21:688. doi: 10.1186/s12885-021-08397-0 (PMC8194219; doi:10.1186/s12885-021-08397-0)
Supplement: Supplementary file 3 — Additional file 3: Table S1. Primers used for RT-PCR. Table S2. Primary antibodies used for immunocytochemistry and immunohistochemistry. [file 12885_2021_8397_MOESM3_ESM.pdf]

Supplemental Table S1 Primers used for RT-PCR

		Sequence	Product size
G3PDH	F	5'-ACC ACA GTC CAT GCC ATC AC-3'	
	R	5'-TCC ACC ACC CTG TTG CTG TA-3'	382bp
LMP-1	F	5'-GGA ATT CCC TTT CCT CAA CTG CCT TGC T-3'	
	R	5'-AAC CGG TTG CAA GCC TAT GAC ATG GTA ATG-3'	1512bp
LMP-1	F	5'-GGA ATT CCG CAA GCC TAT GAC ATG GTA ATG-3'	
	R	5'-CCA CAA TTG ACG GAA GAG GT-3'	237bp
6E6	F	5'-CGG AAT TCC GAA TAG GAG GGA CCG AAA ACG-3'	
	R	5'-CCG CTC GAG CGG CTA CAG GGT CTG GAG GTT GC-3'	591bp
11E6	F	5'-CGG AAT TCC GAA AAA GAG GAG GGA CCG AAA-3'	
	R	5'-CCG CTC GAG CGG TTG TTT GTC CAC CTT GTC CA-3'	660bp
11E7	F	5'-CCG CTC GAG CGG AGT GGA AGG GTC GTT GCT TA-3'	
	R	5'-GGA ATT CCC CGA CCC CTC ATT TTC TGT A-3'	260bp
16E6	F	5'-GAT GGG AAT CCA TAT GCT CTA-3'	
	R	5'-TCG ACC GGT CCA CCG ACC CCT-3'	240bp
16E7	F	5'-GAA GAT CTT CGA AAC CCA GCT GTA ATC ATG C-3'	
	R	5'-CCG CTC GAG CGG TTA TGG TTT CTG AGA ACA GA-3'	297bp

Supplemental Table S2
Primary antibodies used for immunocytochemistry and immunohistochemistry

Primary antibody		Dilutions
Histone H2A.XS139ph (phospho Ser139)	(Gene Tex)	1: 200
Paxillin	(Gene Tex)	1: 400
GIT1 [N3C2], internal	(Gene Tex)	1: 200
MMP2	(Abcam)	1: 400
PCNA	(Abcam)	1: 50
p65	(Santa Cruz)	1: 50
