# Supplementary material for: Co-expression of low-risk HPV E6/E7 and EBV LMP-1 leads to precancerous lesions by DNA damage
Source: BMC Cancer. 2021 Jun 10;21:688. doi: 10.1186/s12885-021-08397-0 (PMC8194219; doi:10.1186/s12885-021-08397-0)
Supplement: Supplementary file 4 — Additional file 4: Table S3. Primary antibodies used for Western blotting. Table S4. Tumors in nude mice. [file 12885_2021_8397_MOESM4_ESM.pdf]

Supplemental Table S3 Primary antibodies used for Western blotting

primary antibody		Dilutions
β-actin	(Cell Signaling Technology)	1: 1000
IκBα	(abcam)	1: 500
phospho-IκBα	(abcam)	1: 500
p105/p50	(abcam)	1: 1000
p100/p52	(abcam)	1: 500
Rel B	(abcam)	1: 1000
ATM	(Cell Signaling Technology)	1: 1000
ATR	(abcam)	1: 1000
Chk1	(Cell Signaling Technology)	1: 1000
Chk2	(abcam)	1: 1000
GIT1 [N3C2]	(Gene Tex)	1: 1000
N-cadherin	(abcam)	1: 1000
MMP2	(abcam)	1: 1000
Paxillin	(Gene Tex)	1: 1000
AKT1	(abcam)	1: 500
p53	(Cell Signaling Technology)	1: 500
phospho-p53	(Cell Signaling Technology)	1: 1000
pRb	(Cell Signaling Technology)	1: 1000
phospho-RB	(Cell Signaling Technology)	1: 1000

Supplemental Table S4 Tumors in nude mice

	tumors in nude mice
mock	0/15
11E6+LMP-1	0/15
11E7+LMP-1	0/15
16E6+LMP-1	6/6
