# Supplementary material for: Co-expression of low-risk HPV E6/E7 and EBV LMP-1 leads to precancerous lesions by DNA damage
Source: BMC Cancer. 2021 Jun 10;21:688. doi: 10.1186/s12885-021-08397-0 (PMC8194219; doi:10.1186/s12885-021-08397-0)

Supplemental Figure S7A: Original blots shown in Figure 2G

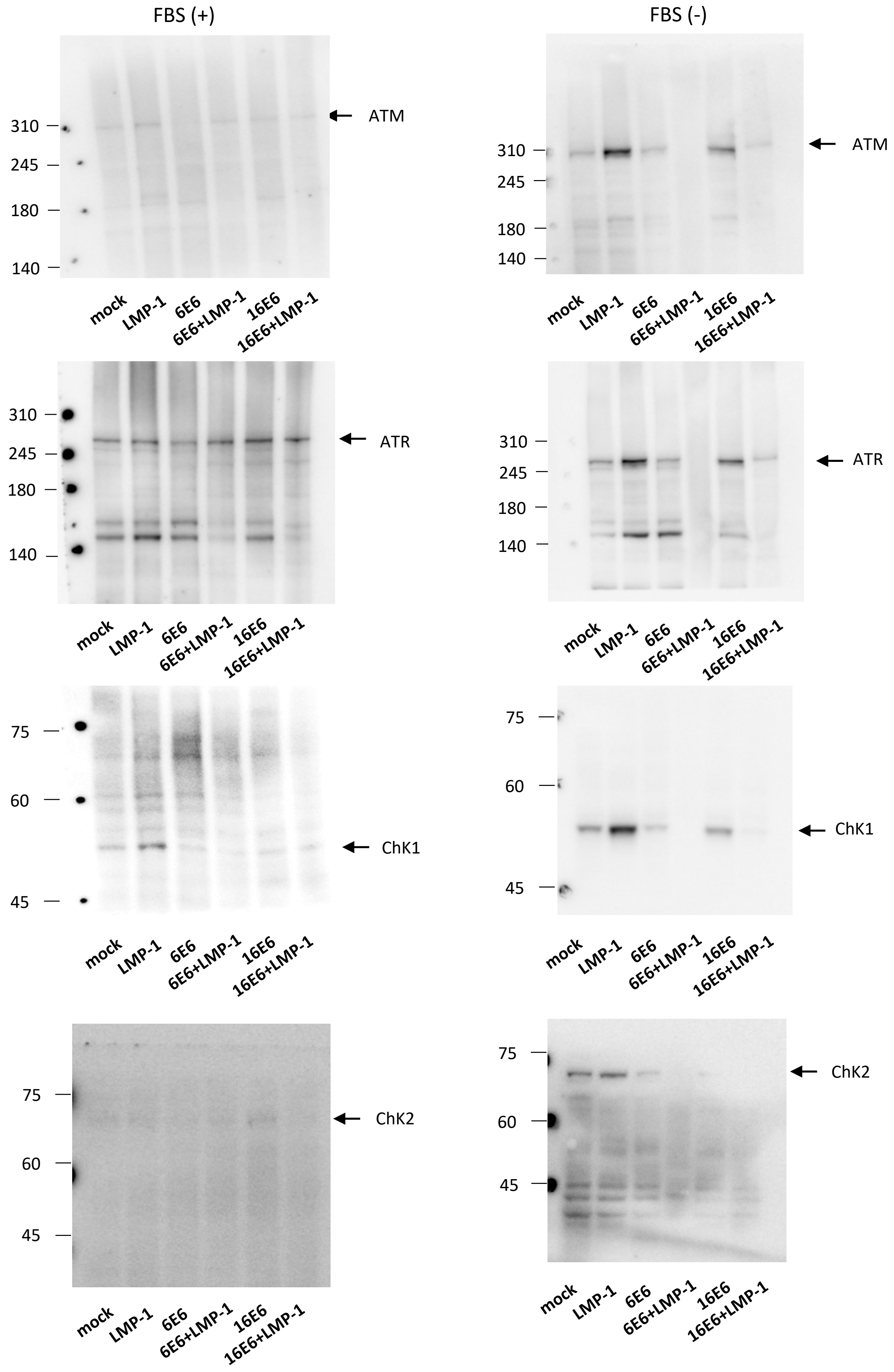

Supplemental Figure S7B: Original blots shown in Figure 2G

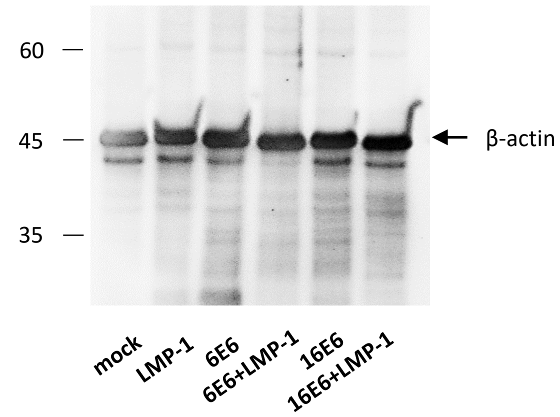

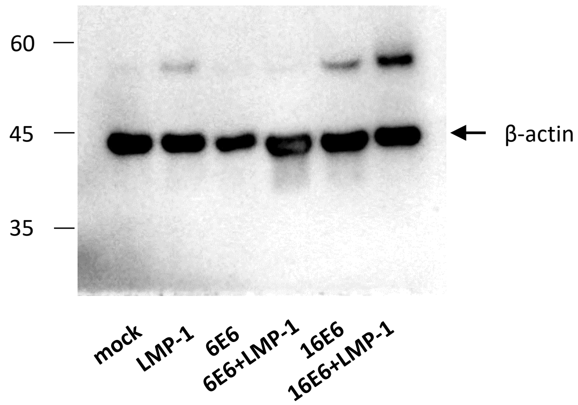

Supplement: Supplementary file 10 — Additional file 10: Figure S7A. Original blots shown in Fig. 2G. Figure S7B. Original blots shown in Fig. 2G. [file 12885_2021_8397_MOESM10_ESM.pdf]
