# Supplementary material for: Co-expression of low-risk HPV E6/E7 and EBV LMP-1 leads to precancerous lesions by DNA damage
Source: BMC Cancer. 2021 Jun 10;21:688. doi: 10.1186/s12885-021-08397-0 (PMC8194219; doi:10.1186/s12885-021-08397-0)

Supplemental Figure S9A: Original blots shown in Supplemental Figure S2E

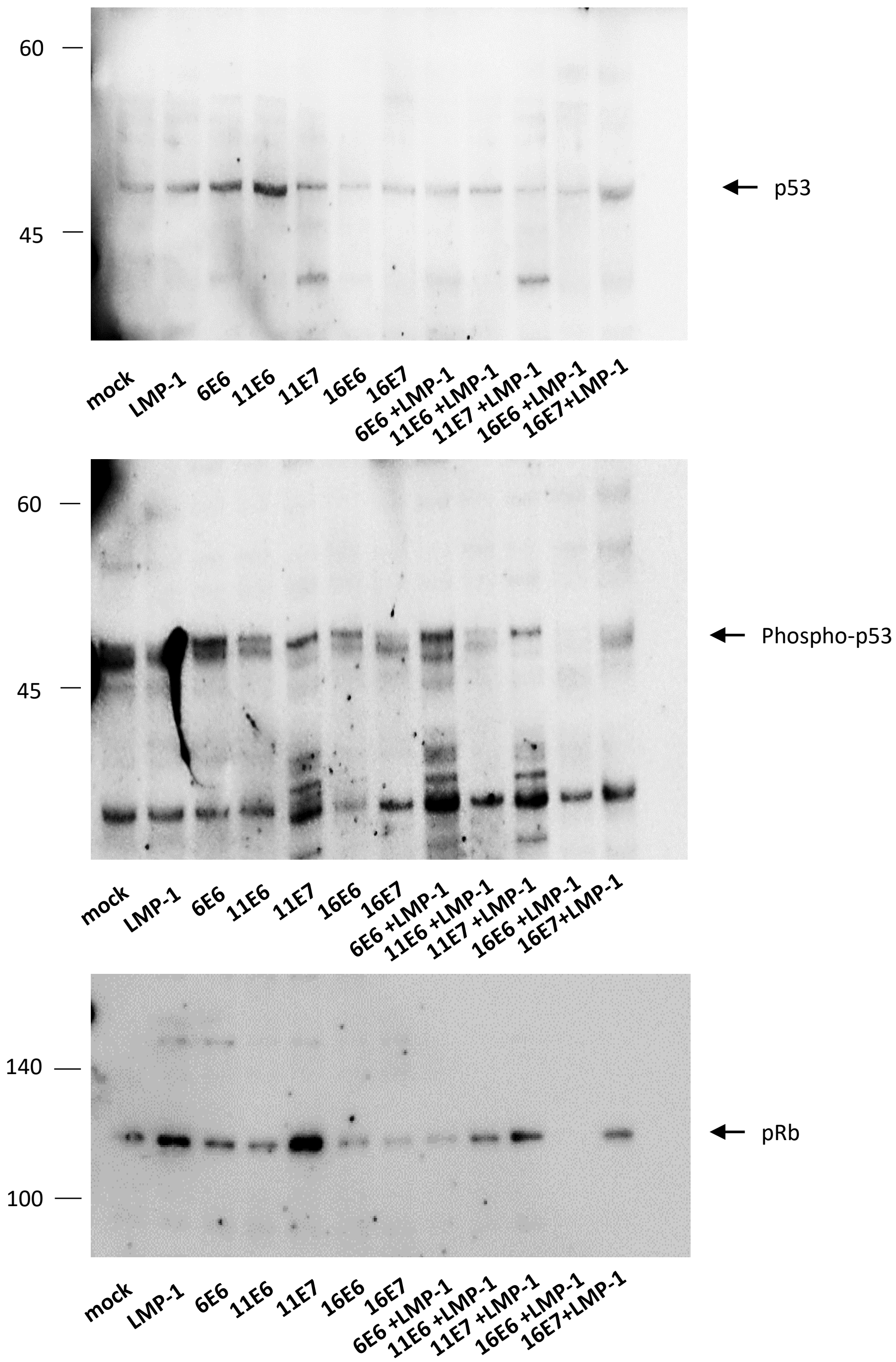

Supplemental Figure S9B: Original blots shown in Supplemental Figure S2E

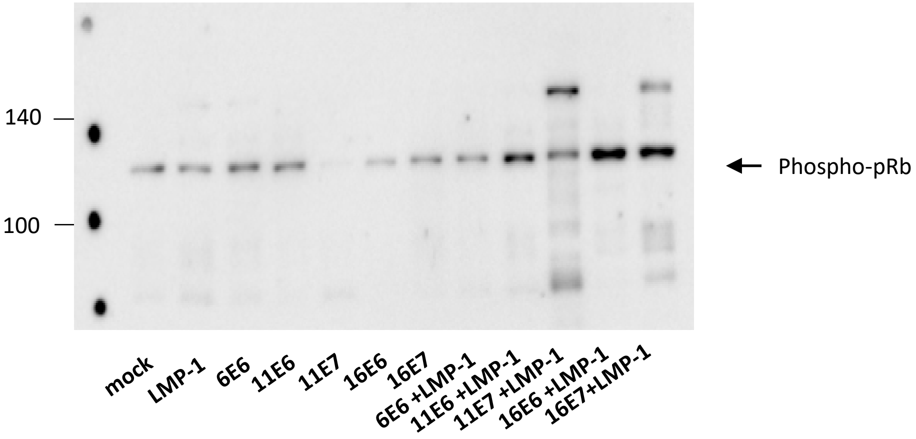

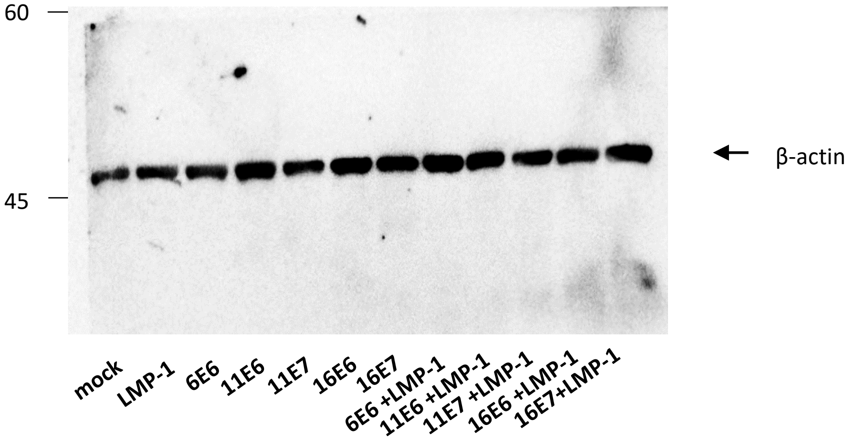

Supplement: Supplementary file 12 — Additional file 12: Figure S9A. Original blots shown in Supplemental Figure S2E. Figure S9B. Original blots shown in Supplemental Fig. S2E. [file 12885_2021_8397_MOESM12_ESM.pdf]
